# Identification and In Vitro and In Vivo Characterization of KAC-50.1 as a Potential α-Synuclein PET Radioligand

**DOI:** 10.1021/acschemneuro.4c00493

**Published:** 2024-11-11

**Authors:** Dinahlee Saturnino Guarino, Patricia Miranda Azpiazu, Dan Sunnemark, Charles S. Elmore, Jonas Bergare, Markus Artelsmair, Gunnar Nordvall, Anton Forsberg Morén, Zhisheng Jia, Miguel Cortes-Gonzalez, Robert H. Mach, Kyle C. Wilcox, Sjoerd Finnema, Magnus Schou, Andrea Varrone

**Affiliations:** † Department of Radiology, Perelman School of Medicine, University of Pennsylvania, Philadelphia, Pennsylvania 19104, United States; ‡ Department of Clinical Neuroscience, Centre for Psychiatry Research, Karolinska Institutet and Stockholm Health Care Services, BioClinicum, Floor 4, Akademiska Stråket 1, 17174 Solna, Sweden; § Offspring Biosciences, Sweden AB, SE-151 36 Södertälje, Sweden; ∥ Applied Immunology, Department of Clinical Neuroscience, Karolinska Institutet, 171 77 Stockholm, Sweden; ⊥ Isotope Chemistry, Drug Safety and Metabolism, AstraZeneca, Pepparedsleden 1, SE-431 83 Mölndal, Sweden; # AlzeCure Pharma AB, Hälsovägen 7, SE-141 57 Huddinge, Sweden; ¶ AbbVie Inc, 1 N Waukegan Road, North Chicago, Illinois 60064, United States; ∇ AstraZeneca, Precision Medicine Diagnostic Development and HBS Science, AstraZeneca R&DRINGGOLD Oncology, KI-RCF PET, J2:30, BioClinicum, Visionsgatan 4, SE-17164 Solna, Sweden

**Keywords:** α-synuclein, Parkinson’s disease, autoradiography, screening assays, PET tracer

## Abstract

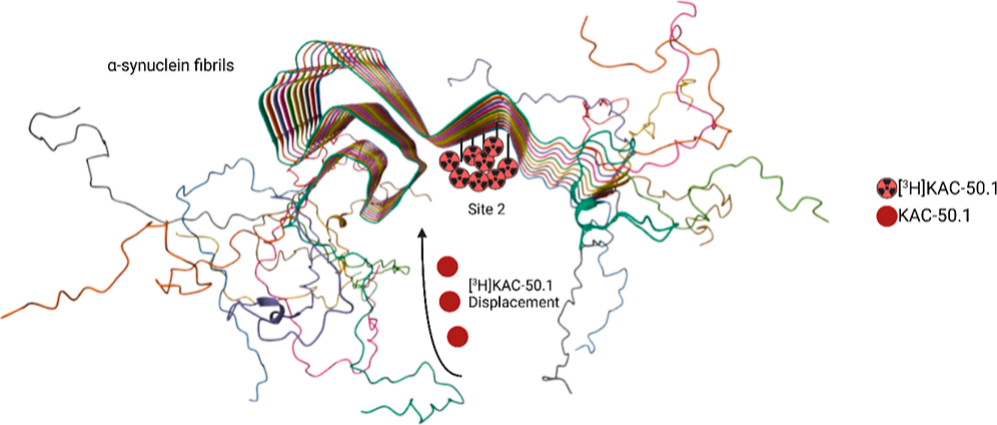

The accumulation of aggregated α-synuclein (α-syn) is a pathological hallmark of Parkinson’s disease (PD) and other synucleinopathies. Here within, we report the in vitro characterization targeting site 2 of α-syn fibrils and in vivo evaluation of NHPs of KAC-50.1 as a potential α-syn positron emission tomography (PET) radioligand. Preclinical studies were performed using a multidimensional approach of post-mortem brain imaging techniques, radioligand binding, and biochemical studies. These experiments were followed by PET imaging in cynomolgus monkeys using [^11^C]KAC-50.1. [3H]KAC-50.1 displayed a KD of 35 nM toward site 2 in recombinant α-syn fibrils. Specific binding of [3H]KAC-50.1 was observed in brain tissues with abundant α-syn pathology but also in AD, PSP, and CBD cases, indicating binding to amyloid β (Aβ) and tau pathology. PET studies showed a rapid entrance of [^11^C]KAC-50.1 into the brain and relatively rapid washout from cortical brain regions, with slower washout in subcortical regions. [3H]KAC-50.1 is a ligand that binds to fibrillar α-syn but shows limited selectivity for α-syn versus Aβ and tau fibrils. PET studies in NHPs indicate that [^11^C]KAC-50.1, despite reversible kinetic properties, displays retention in white matter. Altogether, the in vitro and in vivo properties do not support further development of [^11^C]KAC-50.1 as a PET imaging agent.

## Introduction

Alpha-synuclein (α-syn) aggregates are linked to several neurodegenerative disorders, including Parkinson’s disease (PD), dementia with Lewy bodies (DLB), and multiple system atrophy (MSA). To date, two positron emission tomography (PET) radioligands, [^18^F]ACI-12589 and [^18^F]SPAL-T06, have shown promising in vivo results in MSA patients.^1,2^ The development of an α-syn PET tracer is more challenging in comparison to the development of tracers for other amyloids, such as Aβ or tau. A key reason for this is that the absolute concentration of α-syn aggregates within the brain is thought to be 10- to 50-fold lower than that of Aβ or tau aggregates,^3,4^ and consequently, high affinity of the putative tracer, in the single-digit/subnanomolar range, is required. Next, coexistence and colocalization of α-syn with Aβ and tau aggregates require a PET tracer with high selectivity over Aβ and tau to be able to image α-syn.^5^ Knowledge of the structures of α-syn filaments and how they form may be used for the development of specific biomarkers for synucleinopathies and the development of safe and effective mechanism-based therapies. In 2018, Hsieh et al. conducted a molecular blind docking study using the structural data obtained from the ssNMR study.^6^ This study aimed to identify possible binding regions of small molecules toward α-syn fibrils. The docking studies revealed three putative binding sites, binding sites 2 (Y39-S42-T44; BS2), 9 (G86-F94-K96; BS9), and 3/13 (K45-V48-H50 and K43-K45-V48-H50; BS3/13) and a different selectivity of the classes of compounds used among the binding sites. [^3^H]Tg-1-90B showed a preference for site 2 with a *K*_d_ value of 47 nM for recombinant α-syn fibrils when evaluated in homologous competition studies. In this work, our goal was to identify a compound, using Tg-1-90B as a reference α-syn radioligand, with a higher affinity to α-syn aggregates, higher selectivity toward pathological α-syn compared to other targets in the brain, and favorable pharmacokinetic properties. From a series of compounds synthesized based on available structural information on ligands binding to protein aggregates, a compound, KAC-50.1, was selected based on in vitro screening using recombinant α-syn fibrils and brain tissue microarrays (TMAs). KAC-50.1 was radiolabeled with tritium ([^3^H]) and evaluated with in vitro binding assays using recombinant α-syn fibrils and autoradiography (ARG) studies on post-mortem human brain samples from neurodegenerative disorders and healthy subjects (controls). Brain uptake and washout were assessed by PET in nonhuman primate (NHP) studies.

## Results

### [^3^H]KAC-50.1 Saturation Study with α-Syn Fibrils and in Brain Homogenates from Amyloid-Positive Donors

**Figure 1:**
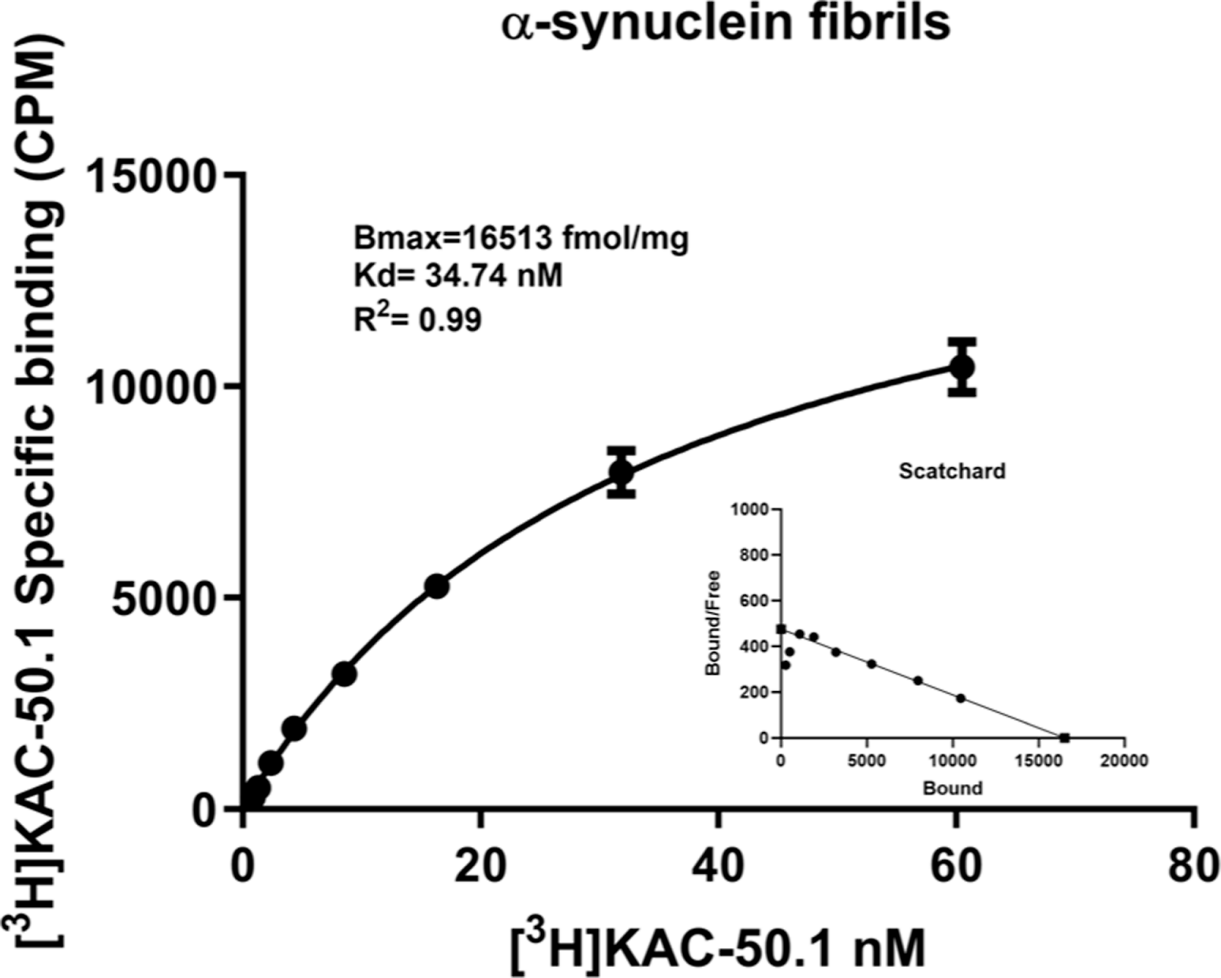
[^3^H]KAC-50.1 saturation binding assay using α-syn fibrils. [^3^H]KAC-50.1 saturation binding assays were carried out in α-syn fibrils using concentrations of 0.8–60 nM. NSB binding was determined using 10 μM of unlabeled KAC-50.1. Scatchard plots indicate *B*_max_ and *K*_D_ values. Error bars represent the mean ± SD for one experiment performed in quadruplicates. *B*_max_ = maximum number of binding sites; *K*_D_ = dissociation constant; and *R*^2^ = regression coefficient.

Saturation binding assay of [^3^H]KAC-50.1 using 500 nM recombinant α-syn fibrils displayed a dissociation constant (*K*_D_) of 35 nM (Figure 1). The formation of α-syn fibrils was confirmed by adding to the solution of fibrils Thioflavin T (ThT) to a final concentration of 10 μM and evaluation with fluorescence correlation spectroscopy as well as confocal laser scanning microscopy (Figure S6). In frontal cortex homogenates from Braak IV donors validated by IHC to be free from tau or α-syn pathology but contain abundant amyloid plaques, the measured *K*_D_ of [^3^H]KAC-50.1 was 5 nM, similar to the *K*_D_ of [^3^H]PiB (Figure S7). However, the *B*_max_ of [^3^H]KAC-50.1 was three times higher than the *B*_max_ of [^3^H]PiB (Figure S7).

### Autoradiography of [^3^H]KAC-50.1 Using TMAs

**Figure 2:**
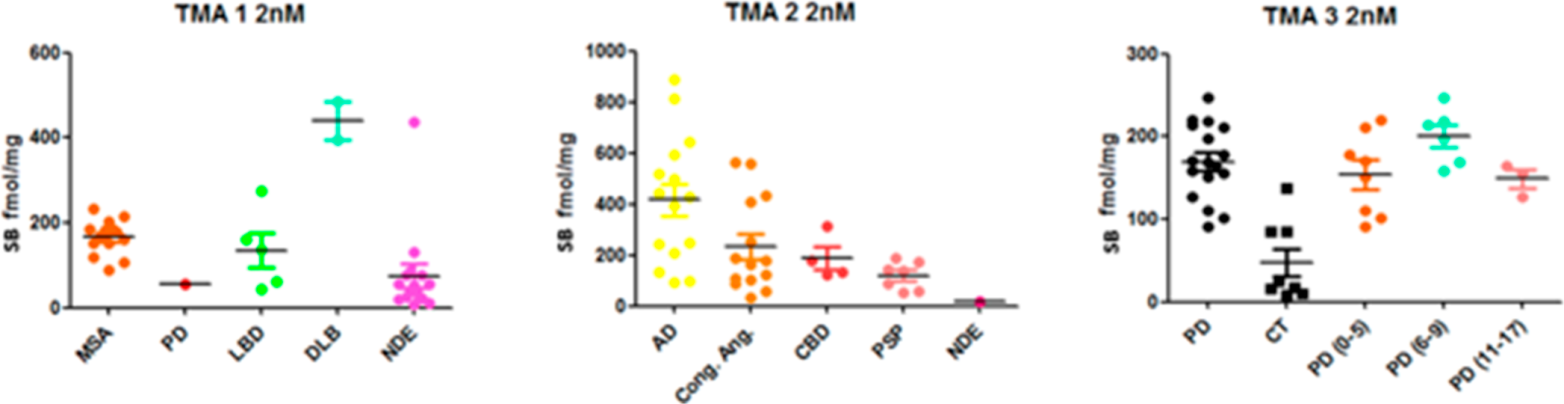
Assessment of specific binding of [^3^H]KAC-50.1 to α-syn and Aβ and tau aggregates in human tissue microarrays sections. In the three autoradiograms, [^3^H]KAC-50.1 total binding is depicted in several neurodegenerative disorders.

**Figure 3:**
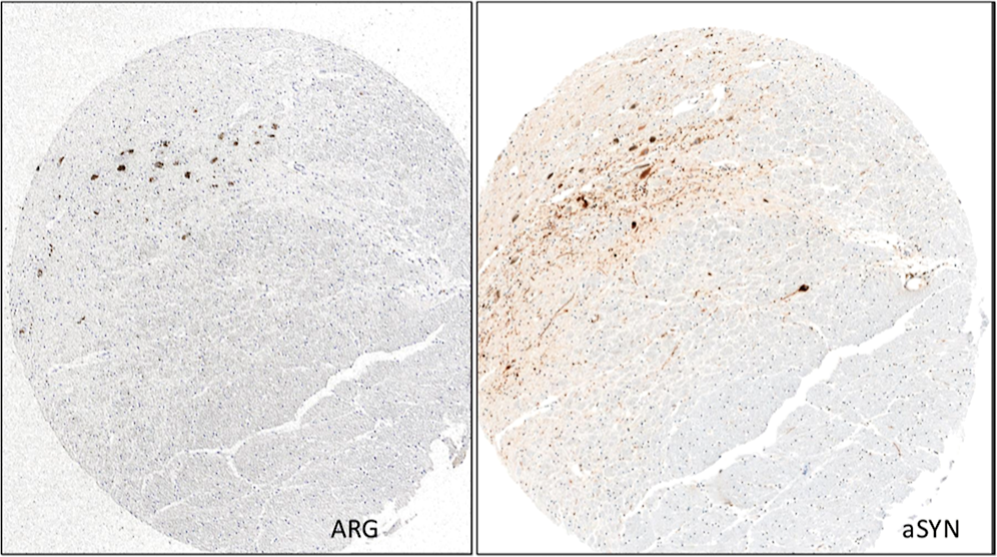
Micro-ARG in Substantia nigra brain section from a PD donor with [^3^H]KAC-50.1 revealed accumulation of silver grains on Lewy bodies but not Lewy Neurites.

**Figure 4:**
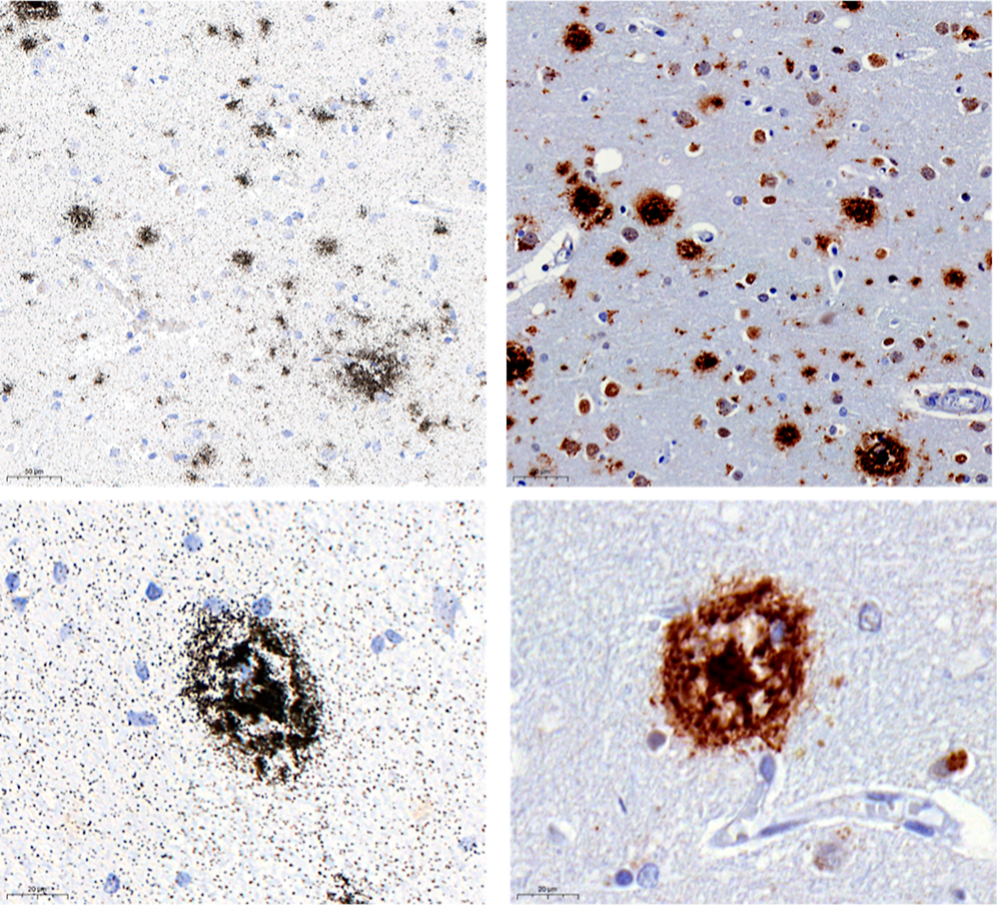
Micro-ARG in brain section from an AD donor with [^3^H]KAC-50.1 revealed accumulation of silver grains on Aβ plaques.

To assess the binding of [^3^H]KAC-50.1 to α-syn pathology and other misfolded proteins, autoradiography using TMAs was performed with three different concentrations of radioligand: 2 nM, 1 nM, and 0.4 nM. The autoradiogram for TMA3 showed that the 2 nM concentration was able to provide the highest binding signal and signal-to-noise ratio (Figures S8–S11). Autoradiography in TMA1 including MSA, PD, Lewy bodies disease (LBD), DLB, and nondemented controls (CT) displayed binding in all cases. Autoradiography in TMA2 including AD, congophilic angiopathy, corticobasal degeneration (CBD), progressive supranuclear palsy (PSP), and controls displayed the highest specific binding in tissue with amyloid-β and lower specific binding in tissues from tauopathies. In TMA3, higher specific binding in PD cases than in the controls was observed. The specific binding was similar in cases with low (0–5), medium,^6−9^ or high level of pathology^10−12^ (Figure 2). Autoradiography results were compared with the immunostaining of adjacent brain sections using α-syn-specific antibodies. The immunostaining of a PD case negative for Aβ and tau, labeling Lewy Bodies, colocalized with the micro-ARG signal of [^3^H]KAC-50.1 in the same section, suggesting target engagement primarily with large Lewy bodies but not with Lewy neurites (Figure 3). In AD cases, the ARG signal of [^3^H]KAC-50.1 corresponded to Aβ (Figure 4).

### In Vitro Autoradiography in Fresh Frozen Tissue

**Figure 5:**
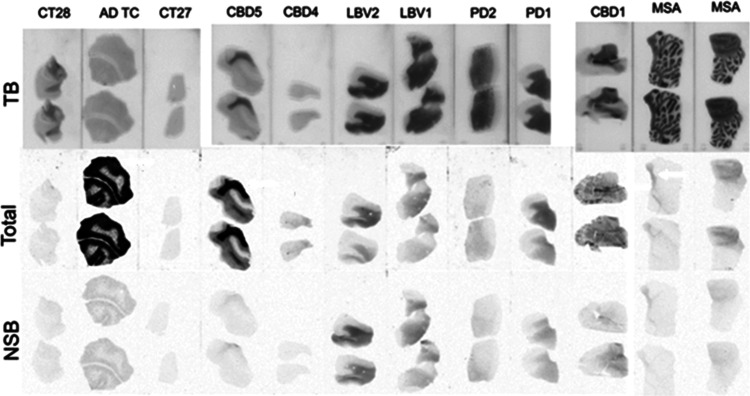
Autoradiogram using 2 nM of [^3^H]KAC-50.1 in fresh frozen tissues. Total binding (middle), nonspecific binding (NSB) determined after coincubation in the presence of 10 μM of KAC-50.1 (bottom), and Toluidine blue (TB) staining (top) are depicted for each slice tissue.

The autoradiography results for the regional distribution of [^3^H]KAC-50.1 are presented in Figure 5. [^3^H]KAC-50.1 displayed specific binding in the white matter of tissue sections from LBV and PD donors, which is most likely to be off target binding and high binding in the gray matter of tissue sections from AD and white matter of CBD donors. Intermediate level of specific binding was observed in the white matter of tissue sections from MSA donors.

### Radiochemistry

The synthesis, purification, and formulation of [^11^C]KAC-50.1 were accomplished in 35–40 min from the end of the irradiation and afforded 782 ± 60 MBq (*n* = 2) with a radiochemical purity of >95%. The product was identified by coinjection with a reference standard. The molar activity of the formulated product at the end of the synthesis was 482–937 GBq/μmol.

### [^11^C]KAC-50.1 PET Studies in NHPs

**Figure 6:**
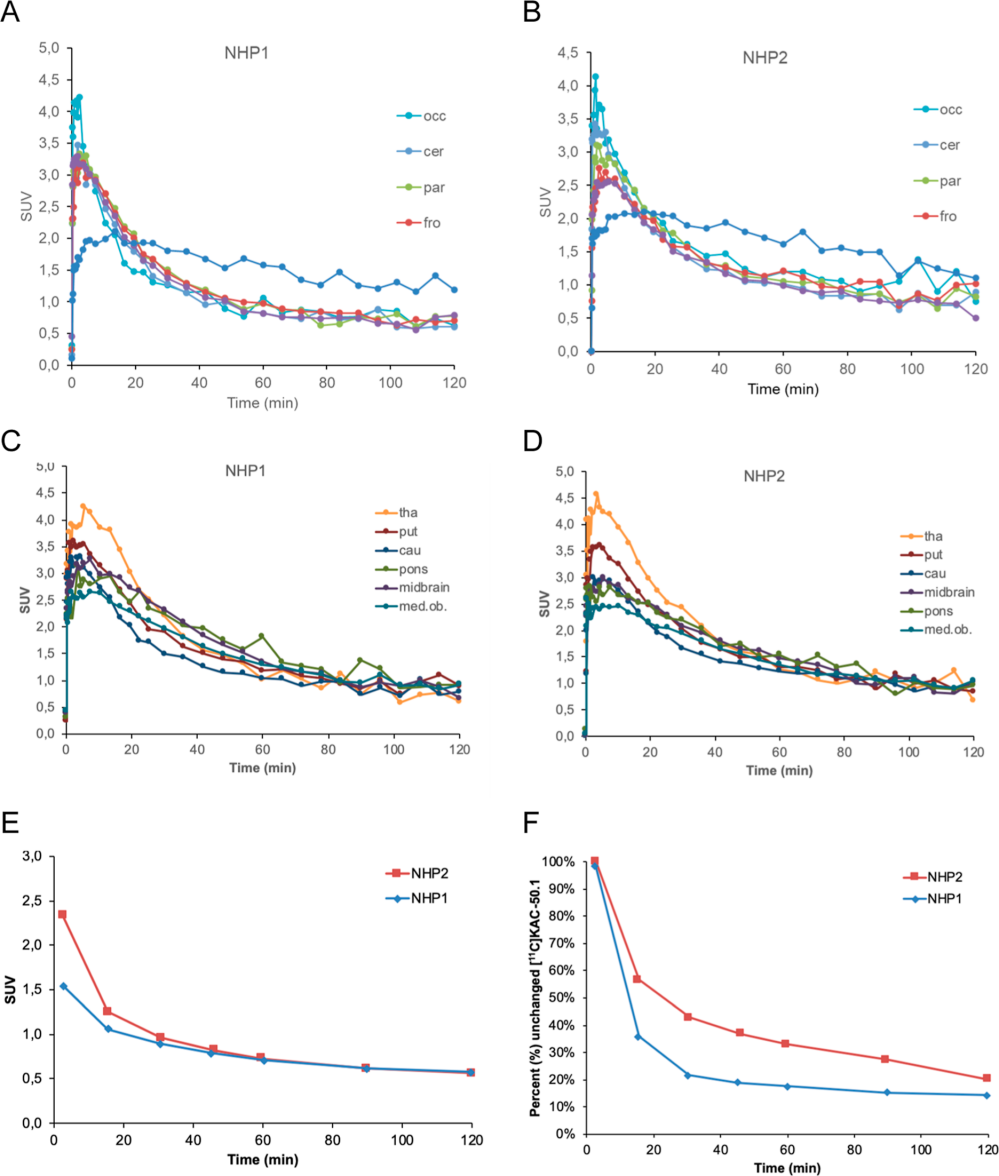
Brain time activity curves of [^11^C]KAC-50.1 in cortical regions and white matter (A,B) and in subcortical regions (C,D) in the two cynomolgus monkeys. Blood radioactivity (E) and unchanged fraction of [^11^C]KAC-50.1 in the plasma (F).

After intravenous injection in two NHPs, [^11^C]KAC-50.1 rapidly entered the brain with a *t*_max_ = 3.5 min and a peak SUV ∼ 2.9 (Figure S10). The washout from the brain was moderately fast with a *C*_max_/*C*_60 min_ = 2.4–2.7 and a *C*_max_/*C*_120 min_ = 3.4–3.7. Slower washout was observed in the white matter (Figure 6A,B) and was more rapid in the cortical regions and cerebellum (Figure 6A,B) as compared with subcortical regions (Figure 6C,D). The elimination and metabolism of [^11^C]KAC-50.1 was relatively fast, with 20–40% unchanged radioligands present in the plasma at 60 min after injection (Figure 6F).

## Discussion

In this study, we explored the in vitro and in vivo binding properties of the KAC-50.1 compound, whose selection was based on ARG-based competition assays performed in the presence of Tg1-90B, an α-syn ligand developed by the Michael J Fox foundation (MJFF) consortium and that showed binding to site 2 of α-syn fibril.^6^ In vitro binding assays using recombinant fibrils were performed to evaluate the binding affinity and the maximum number of binding sites (*B*_max_) of the compound to α-syn. The use of α-syn fibrils is helpful for large-scale screening of compounds, but the characterization in brain tissue at an early stage is necessary to understand the property of candidate leads. Therefore, further characterization was done with autoradiography using human post-mortem fresh frozen tissues and paraffin-embedded tissue microarrays. The affinity of [^3^H]KAC-50.1 measured using recombinant fibrils was in the range of 34 nM, but it was not possible to confirm this result in brain homogenates due to high values of the nonspecific binding (NSB). Nevertheless, the reason for the high NSB remains not fully understood. Characterization of [^3^H]KAC-50.1 by ARG using TMAs confirmed the presence of specific binding to Lewy bodies found in the Substantia nigra from PD cases. However, specific binding was observed in tissues from AD and CBD cases, indicating binding to amyloid-β and tau pathology. Interestingly, the *K*_D_ of [^3^H]KAC-50.1 measured in brain homogenates containing pure amyloid pathology was similar to the K_D_ of [^3^H]PiB (5.5 nM), although the *B*_max_ (4675 fmol/mg) in Figure S7 was much higher. A possible explanation is that [^3^H]KAC-50.1 binds to multiple sites on the Aβ fibrils. Although established PET radioligands for Aβ are available and no further developments are on the way, it is possible that [^3^H]KAC-50.1 might be more sensitive than PiB in detecting a small amount of Aβ in the brain. We also observed remarkable specific binding of [^3^H]KAC-50.1 in tissue sections from CBD cases, suggesting that the radioligand also binds to 4R tau. The affinity of [^3^H]KAC-50.1 for 4R tau was not further investigated since it was not the main objective of this study. Finally, the nature of the displaceable binding of [^3^H]KAC-50.1 observed in the control tissues was not clarified. Despite the lack of success in identifying a selective α-syn radioligand, we believed that the approach used in this study shows that TMAs are a useful tool for the evaluation of ligands in tissue from different proteinopathies, using autoradiographic assays. NHP PET studies showed a good brain uptake of [^11^C]KAC-50.1 and a faster washout in the cortical regions compared to white matter. The retention of [^11^C]KAC-50.1 in white matter is probably related to the lipophilicity of the ligand, but we cannot exclude displaceable binding to the white matter. Although the radioligand binds to α-syn fibrils in vitro and displays specific binding in tissue with α-syn, its low affinity and limited selectivity are not suitable for further development as a PET imaging agent.

## Materials and Methods

### Radiosynthesis of [^3^H]Tg-1-90B

The precursor and standard of [^3^H]Tg-1-90B were provided by Prof. Robert Mach. [^3^H]Tg-1-90B was synthesized from the desmethyl precursor using [^3^H]CH_3_I and according to the method previously described.^7^ The final concentration was 2.65 mCi/mL, with a molar activity of 47 mCi/μmol. The radiochemical purity was greater than 98%.

### Radiosynthesis of [^3^H]KAC-50.1

**Figure 7:**
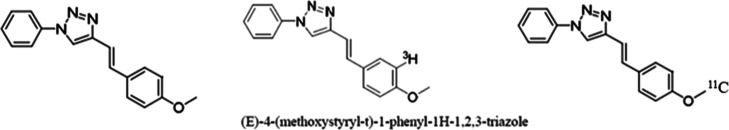
Chemical structure of KAC-50.1 and [^3^H]/[^11^C]KAC-50.1.

Tritium labeling of KAC-50.1 (Figure 7) was carried out at AstraZeneca. Analysis and purification were performed by high-performance liquid chromatography (HPLC) on an ACE of 3.5 μm C18, 6 mm × 100 mm column. The product was eluted by a mixture of acetonitrile (MeCN) and water +0.1 M formic acid at a flow rate of 1 mL/min. The effluent from the column was monitored for UV absorbance (254 nm) and radioactivity. Afterward, it was dissolved into a mixture of water and ethanol, and it resulted in a stock solution with a concentration of 1.29 mCi/mL and a molar activity of 25 Ci/mmol. The radiochemical purity was greater than 99.0%, and the chemical purity was greater than 50.0%.

### Radiosynthesis of [^11^C]KAC-50.1

[^11^C]CH_4_ was obtained from a GEMS PETtrace cyclotron (GE Healthcare, Uppsala, Sweden) via the ^14^N(p,α)^11^C nuclear reaction. The target gas (10% hydrogen in nitrogen) was irradiated using 16.4 MeV protons at 35 μA for 20–30 min. The generated [^11^C]CH_4_ was released from the target and directed to a TracerMaker synthesis module (ScanSys Laboratorieteknik ApS, Copenhagen, Denmark) where it was trapped in a HayeSep trap at −180 °C. The concentrated [^11^C]CH_4_ was transformed into [^11^C]CH_3_I by recirculation with I_2_ vapors in a quartz-glass oven at 720 °C. The synthesized [^11^C]CH_3_I was concentrated in the HayeSep trap at −10 °C. The concentrated [^11^C]MeI was released at 210 °C in a stream of helium into a column containing AgOtf at 200 °C to produce [^11^C]MeOTf. The produced [^11^C]MeOTf was directly trapped in a reaction vial containing the des-methylated precursor of KAC-50.1 (3.0 mg) and NaOH (0.5 M in H_2_O, 4 μL) in acetone (500 μL) at room temperature. The mixture was allowed to react at room temperature for one min before being diluted with water and injected into a semipreparative HPLC column for purification. The purification was performed on a XBridge BEH C18 OBD preparative column (130 Å, 5 μm, 10 mm × 250 mm, Waters) using acetonitrile and aqueous ammonium formate (0.1 M) at a 54:46 ratio at 6 mL/min monitoring the effluent for radioactivity and UV absorbance. The fraction containing [^11^C] KAC-50.1 was collected and diluted with water (50 mL) and concentrated using a SPE cartridge (Sep-Pak tC18 1 cm^3^ Vac Cartridge, Waters). After washing the SPE cartridge with water, the product was eluted using ethanol (0.8 mL) into a vial containing a solution of Tween-80 (0.8%) in phosphate-buffered saline (5 mL) prior to sterile filtration into a vial containing phosphate-buffered saline (3 mL). The chemical and radiochemical purity as well as the identity of [^11^C] KAC-50.1 was assessed by analytical HPLC (Agilent G1314D, G1311A, and G1322A in series with a radiodetector) using a XBridge C18 column (130 Å, 3.5 μm, 4.6 × 150 mm, Waters) and a mobile phase of acetonitrile and aqueous ammonium formate (0.1 M) at a 50:50 ratio and a flow of 3.0 mL/min, monitoring the effluent for radioactivity and UV absorption (254 nm). The synthesis, purification, and formulation of [^11^C]KAC-50.1 was accomplished in 35–40 min from the end of the irradiation and afforded 782 ± 60 MBq (*n* = 2) with a radiochemical purity of >95%. The product was identified by coinjection with a reference standard. The molar activity of the formulated product at the end of the synthesis ranged 482–937 GBq/μmol.

### Autopsy Material

Frozen human brain tissues from Lewy body disease (LBD), PD, Alzheimer’s disease (AD), and control patients were obtained from The Netherlands brain bank (NBB) (Table 1) and the UK brain bank (UKBB) (Table 2).

**Table 1:** Demographics of the Fresh Frozen Tissues Included in the Study, from the NBB

NBB	code	diagnosis	brain region	age	gender	IHC
	CD4	corticobasal degeneration	superior frontal gyrus	58	F	pure tau pathology
	CD5	CBD	superior parietal gyrus	58	F	pure tau pathology
	LB1	LBD	cingulate gyrus	54	M	pure α-syn pathology
	LB2	LBD	cingulate gyrus	83	M	pure α-syn pathology
	PD1	PD	cingulate gyrus	69	M	pure α-syn pathology
	PD2	PD	cingulate gyrus	77	M	pure α-syn pathology
	AD	Alzheimer’s disease	parietal cortex	80	F	mixed tau and A-β
	CT24	nondemented control	cingulate gyrus	79	F	negative
	CT27	nondemented control	cingulate gyrus	81	F	negative
	CT28	nondemented control	cingulate gyrus	51	M	negative

**Table 2:** Demographics of the Fresh Frozen Tissues Included in the Study, from the UK Brain Bank^a^

UKBB	code	diagnosis	brain region	Abeta (6E10/4G8)	pTau (AT-8)	a-SYN (LB509)
	MSA 1	MSA	superior frontal gyrus	0	0	2
	MSA 1	MSA	putamen	0	0	2
	MSA 2	MSA	superior frontal gyrus	0	0	2
	MSA 2	MSA	putamen	0	1	2.5
	MSA 3	MSA	putamen	0	0	3

a The score 1–3 refers to a qualitative assessment of the amount on pathological inclusions.

**Figure 8:**
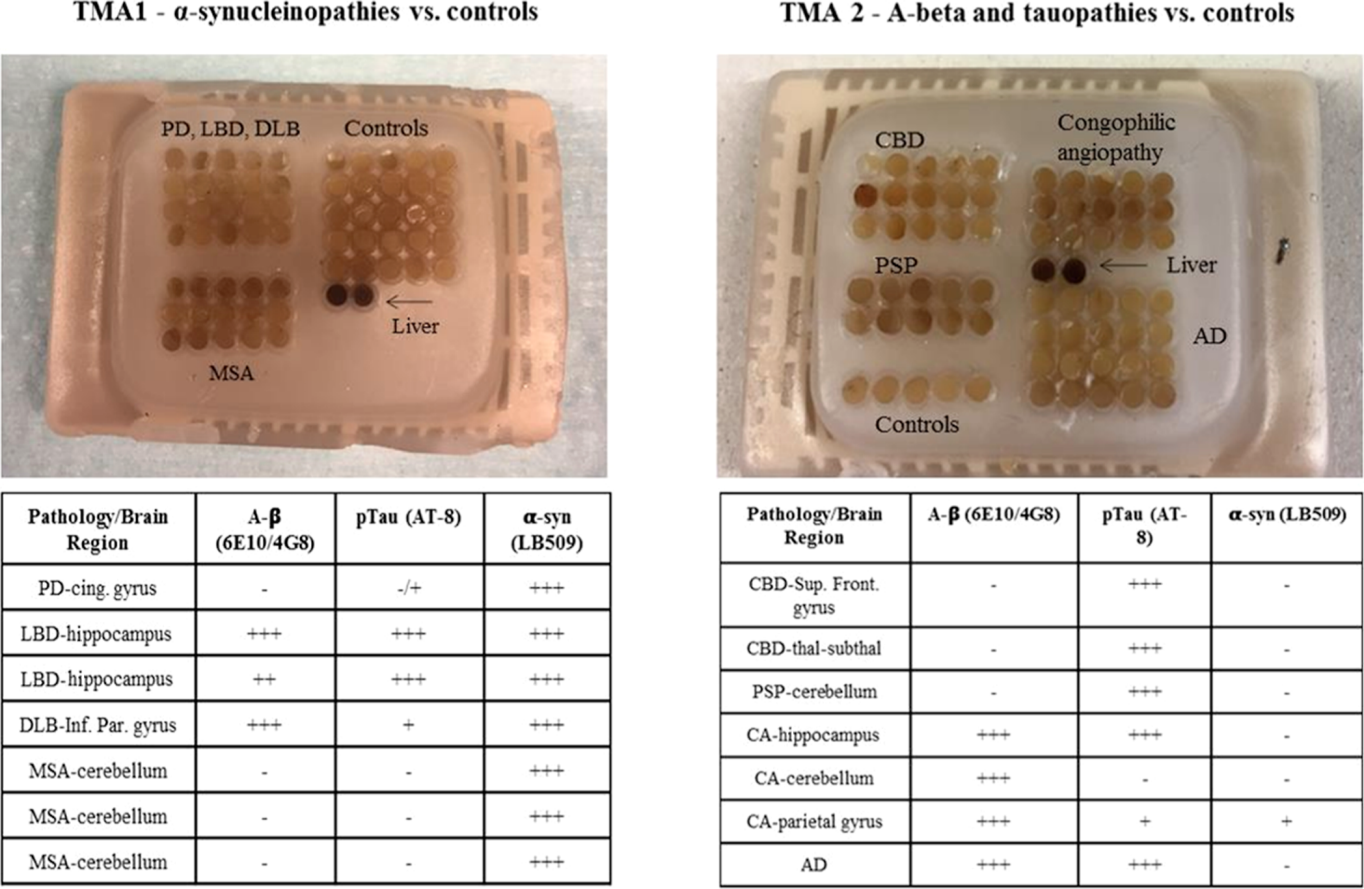
Tissue microarrays 1 and 2, containing the cases previously described; in the table is reported the amount of Aβ, tau, and α-syn deposits for each of the samples. The score + to +++ refers to a qualitative assessment of the amount of pathological inclusions.

All tissue blocks were stained for Aβ (6E10/4G8), α-syn (LB509), and phospho-tau (AT8) immunoreactivity. Abeta, α-syn, and tau loads were assessed by a qualitative analysis, and based on their burden, brain sections were divided into three groups: (1: low, 2: moderate, and 3: high score of pathology). Additionally, three types of tissue-microarrays (TMAs) were generated from a series of paraffin-embedded tissue blocks from different brain regions of neurodegenerative diseases: TMA1 (Figure 8) contained samples from PD, LBD, DLB, MSA, and nondemented controls and TMA2 (Figure 8) donors from cerebral amyloid angiopathy (CAA), PSP, Alzheimer’s disease (AD), and CBD.

**Figure 9:**
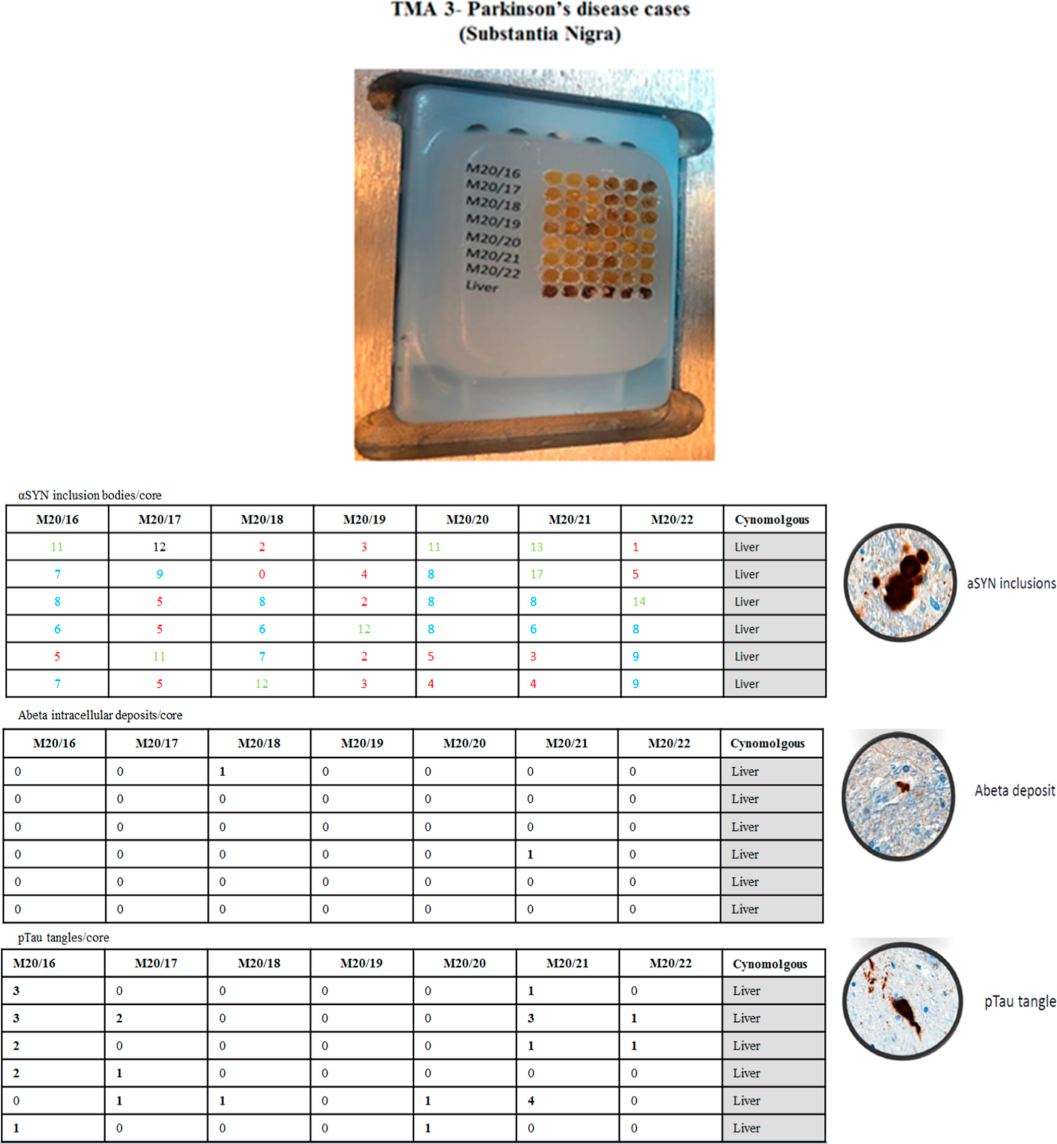
Representation of tissue microarray 3 (TMA3), containing tissue from the substantia nigra of PD and nondemented control subjects (not depicted in the picture); in the tables is reported the score values for α-syn, Aβ, and tau deposits; in the first table, the values in red represent low content of α-syn, in blue mild content, and in green high content.

Finally, TMA3 was generated from a series of paraffin-embedded tissue blocks of the substantia nigra from patients with PD and controls (Figure 9).

### α-Syn Fibrils

α-Syn was provided by Umeå University. The construct for human α-syn was used from GenScript (NJ, USA) and cloned into a pET-3a vector according to the previously published procedure.^7^ For the fibril formation, 2 mg of lyophilized stock solution of α-syn was dissolved in 1 mL of PBS plus salt buffer at pH 7.4 containing 0.15% of BSA. The concentration of α-syn in the stock was 70 μM. The vial was incubated at 37 °C for 9 days using constant shaking at 450 rpm. For determination and quantification of α-syn fibrils, ThT assay was carried out. All samples contained 20 μM ThT, and fluorescence was measured at 480 nm (excitation 440 nm) every 10 min. The final α-syn concentration on each well for binding experiment was 1 μM.

### In Vitro Binding Assays and Competition Studies Using TMAs

The identification of KAC-50.1 was performed through a combination of in vitro binding assays and competition studies using TMAs. Details of the binding assays and competition studies are described in the Supporting Information. In these experiments, [^3^H]Tg-1-90B was used as a reference α-syn radioligand based on the knowledge that the ligand binds to site 2 on α-syn fibrils.^6^ A library of compounds was designed based on the structure of Tg-1-90B: 20 compounds were selected from an internal library at AstraZeneca and 18 compounds were custom-designed. From the initial screening using α-syn fibrils, compounds that inhibited 75% or more of the total binding of [^3^H]Tg-1-90B to α-syn fibrils at a concentration of 300 nM were selected (Figure S3). Nine compounds fulfilled those criteria and were further selected for competition experiments on TMAs. From these experiments, three compounds (50.1, 48.1, and 16) were identified that were able to block the binding of [^3^H]Tg-1-90B in TMAs from MSA cases and displayed limited competition in TMAs from cases of AD and CAA (Figures S4 and S5). 50.1 was selected as the first ligand to be labeled with [^3^H] and further evaluated in vitro and in vivo.

### In Vitro Binding Assays

#### Saturation Binding Assay

Saturation binding assays were performed using a [^3^H]KAC-50.1 concentration range of 0.8–60 nM together with α-syn fibrils (500 nM final concentration) in PBS + 0.15% BSA buffer (pH 7.4). The NSB was determined with an excess of unlabeled KAC-50.1 (1 μM) in a total volume of 150 μL. The binding mixture was incubated at 37 °C for 2 h shaking 450 rpm in 96-well plates. The incubation was stopped by vacuum filtration onto 0.5% PEI-presoaked GF/C filters using a 96-well FilterMate harvester, followed by three washes with cold wash buffer containing 20% EtOH. Filters were dried for few minutes at 35 °C and sealed in polyethylene, the scintillation cocktail (Betaplate Scint; PerkinElmer) was added, and the radioactivity was counted in a Wallac TriLux 1450 MicroBeta counter. At the end of the experiments, laboratory instruments and materials were cleaned by using 1% sodium dodecyl sulfate (SDS) for inactivating pathogenic α-syn.^8^ The saturation data was fitted and analyzed using the nonlinear regression function of GraphPad Prism 10 software to calculate the dissociation constant (*K*_D_) and maximum number of binding sites (*B*_max_). Scatchard plots were prepared with GraphPad Prism 10 software to display the saturation binding data.

### In Vitro Autoradiography Experiment

In vitro autoradiography was performed using fresh frozen and deparaffinized tissue microarray sections derived from MSA, PD, AD, CBD, PSP, and CT brains. Brain sections were first equilibrated for 15 min in 1× PBS and then incubated with 2, 1, and 0.4 nM [^3^H]KAC-50.1 in assay binding buffer 1× PBS + 20% EtOH for 60 min at RT. To determine the NSB, adjacent brain sections were incubated with [^3^H]KAC-50.1 mixed with 10 μM unlabeled KAC-50.1. After incubation, slides were washed 4 times for 10 min in cold washing buffer 1× PBS + 20% EtOH to remove the unbound tracer and then dipped briefly in distillated water. The slides were dried and exposed to the phosphor imaging plates (Fujifilm Plate BASTR2025, Fujifilm, Tokyo, Japan). Tritium microscale standards (American Radiolabeled Chemicals Inc.) were placed in cassettes together with the sections for calibration and quantification of the binding density. For image analysis, the phosphor imaging plates were exposed for approximately 90 h. Then, the films were scanned, and the resulting images were processed with a Fujifilm BAS-5000 phosphor imager (Fujifilm, Tokyo, Japan). Analysis was performed using Multi Gauge 3.2 phosphor imager software (Fujifilm, Tokyo, Japan). Manual delineation of each region of interest (ROI) was performed visually on each digital image using 3- to 4-fold magnification. Mean pixel values of the ROIs from each section were transformed into radioactivity values using the tissue standards used for creating a calibration standard curve. Based on these measurements, specific binding values were calculated in the presence of the inhibitor (total binding—NSB).

### High-Resolution Autoradiography

After incubation and ARG experiment, slides were washed 3 × 10 min in wash buffer at 1 °C, followed by a quick dip in ice-cold ddH_2_O and air-dried and stored under vacuum until dipping in photoemulsion. In the dark, using a sodium lamp, ILFORD Nuclear Emulsion Type K2 emulsion was melted in a heated water bath and diluted (1:1) with ddH_2_O. Emulsion was poured into dipping chambers, and slides were dipped and placed vertically to air-dry. When completely dried, slides were placed in a light-tight slide box with desiccant and exposed at 4C for 1–12 weeks. After exposure, slides were developed in diluted Phenisol Developer for 2 min, rinsed in water, and fixed in hypam Fixer at 17 °C in a water bath. After rinsing in water, slides were counterstained with Harris HTX, dehydrated in graded ethanol, cleared in xylene, and mounted in Pertex. Images were taken using a 3D Histech P250 II scanner at up to 10 focal layers with a distance of 0.2 microns.

### Immunohistochemistry

Immunohistochemical chromogenic (IHC) staining was performed using reference antibodies for respective proteinopathies: Signet laboratories (Abeta 6E10, Abeta 4G8), Abcam (anti-Alpha-synuclein antibody [LB509]), and Thermo Fisher Scientific (AT-8 Phospho-PHF-Tau). Briefly, 5 μm slide-mounted tissue sections were performed according to a standardized protocol with modifications to optimize the specificity of the observed staining patterns. IHC staining was performed using the Discovery Ultra platform (Ventana) automated immunostaining robot, using the OmniMap DAB chromogenic staining kit (Ventana Medical Systems) according to the manufacturer’s instructions. In brief, initial deparaffinization, followed by heat-activated antigen retrieval in a pH 8.0 buffer, was performed to improve the detection of antigens in the FFPE tissue. Endogenous tissue peroxidases (Inhibitor CM, Ventana), which may interfere with the assays, were blocked with 0.3% hydrogen peroxide. The primary antibody for phosphorylated Tau detection (AT-8 (pTau was applied, followed by incubation with the HRP-conjugated secondary antibody (the HPR-labeled OmniMap goat anti-Mouse Ab). Visualization of the positively stained cells was performed by addition of hydrogen peroxide and DAB (single IHC), resulting in an insoluble brown (DAB) staining precipitate at the site of antibody binding. Counterstaining for IHC was done with hematoxylin [Hematoxylin II, Ventana, 760-2208 and Bluing Reagent, Ventana, 760-2037]. The stained slides were subsequently scanned at up to 10 focal layers in brightfield (20× -40 objective) using a digital whole slide scanner [Pannoramic 250 II Scanner, 3DHistech, Budapest, Hungary). Analysis of the IHC-stained tissue sections was performed by manual evaluation, using digital image viewer software (CaseViewer). All analyses of the stained and labeled tissue sections by reference antibody AT-8 and/or ligands were compared, side by side, to each other.

### PET Imaging Experiments in Cynomolgus Nonhuman Primates

The in vivo studies in cynomolgus NHPs were conducted at Karolinska Institutet in Stockholm, Sweden. The NHPs were housed in Astrid Fagraeus Laboratory (AFL), Comparative Medicine, Karolinska Institutet, Solna, Sweden. The research was approved by the Animal Ethics Committee of the Swedish Animal Welfare Agency (Dnr 10367-2019) and was performed according to “Guidelines for planning, conducting, and documenting experimental research” (Dnr 4820/06-600) of Karolinska Institutet.

### Study Details

Anesthesia was induced by intramuscular injection of ketamine hydrochloride (∼10 mg/kg) at AFL and maintained by the administration of a mixture of sevoflurane (2.0–8.0%), oxygen, and medical air with endotracheal intubation at the KI PET center. The NHP head was immobilized with a fixation device. Body temperature was maintained and monitored by an esophageal thermometer. ECG, heart rate, blood pressure, respiratory rate, and oxygen saturation were continuously monitored throughout the experiments. Fluid balance was maintained by a continuous infusion of saline. Each NHP was imaged for 2 h after intravenous bolus injection of [^11^C]KAC-50.1 with a radioactivity of 149 and 162 MBq, respectively. PET imaging was conducted by using a high-resolution research tomograph (Siemens Molecular Imaging). A transmission scan of 6 min using a single ^137^Cs source was performed before the PET measurements. List-mode data were reconstructed into a series of 35 frames (10 s × 4, 20 s × 4, 1 min × 4, 3 min × 7, and 6 min 16). Venous blood sampling was performed manually for the measurement of radioactivity and metabolism at 2.5, 15, 30, 45, 60, 90, and 120 min after the injection.

### Plasma Analysis

Radioactivity in venous blood was measured using a well counter after sampling, as described above. The fraction of plasma radioactivity corresponding to unchanged [^11^C]KAC-50.1 was determined for all time points specified above using gradient HPLC with radiodetection.

### PET Image Analysis

Volumes of interest (VOIs) were delineated on coregistered PET/MR Images using PMOD software (version 3.7; PMOD Technologies). The VOIs encompassed the following regions: Whole brain (WB); Thalamus (Tha); Putamen (Put); Caudate nucleus (Cau); Pons; Midbrain; Medulla Oblongata (med. ob.); Cerebellum (Cer); Occipital cortex (Occ); Parietal cortex (Par); Frontal cortex (Fro); Temporal cortex (Tem); and White matter (WM). Decay-corrected time–activity curves (TACs) for all regions were plotted. Radioactivity concentration was expressed as a standardized uptake value and calculated as radioactivity concentration (kBq/cm^3^)/(radioactivity injected [MBq]/body weight [kg]).

## Supporting Information Available

HPLC and Radio-HPLC plots of [11C]KAC-50.1; in vitro binding screening assay in the presence of α-synuclein fibrils from MSA, AD, and CAA cases of the tissue microarrays (TMA1, TMA2, and TMA3), respectively; α-synuclein aggregation assay using Thioflavin T fluorescence assay; depicting saturation binding studies of [^3^H]KAC 50.1 and [^3^H]PiB in AD brain homogenates; autoradiograms of [^3^H]KAC 50.1 in TMAs 1, 2, and 3 in the presence of 2, 1, and 0.4 nM of radiolabeled compound; specific binding of [^3^H]KAC 50.1 in the TMAs; and NHP PET imaging with [^11^C]KAC-50.1 and corresponding whole-brain time activity curves (PDF)

## Abbreviations

PD: Parkinson’s disease
AD: Alzheimer’s disease
MSA: multiple System Atrophy
CAA: congophilic amyloid angiopathy
TMAs: tissue microarrays
PIB: Pittsburgh compound B
*B*_max_: maximum number of binding sites
*K*_D_: dissociation constant
ThT: Thioflavin T
IHC: immunohistochemistry
TB: Toluidine blue
