# Supplementary material for: Identification and In Vitro and In Vivo Characterization of KAC-50.1 as a Potential α-Synuclein PET Radioligand
Source: ACS Chem Neurosci. 2024 Nov 11;15(22):4210–9. doi: 10.1021/acschemneuro.4c00493 (PMC11587505; doi:10.1021/acschemneuro.4c00493)
Supplement: Supplementary file 1 — cn4c00493_si_001.pdf [file cn4c00493_si_001.pdf]

Supplementary material

Identification, in vitro and in vivo characterization of KAC-50.1 as potential α-synuclein PET radioligand

Dinahlee Saturnino Guarino*, Patricia Miranda Azpiazu, Dan Sunnemark, Charles S. Elmore, Jonas Bergare, Markus Artelsmair, Gunnar Nordvall, Anton Forsberg Morén, Zhisheng Jia, Miguel Cortes-Gonzalez, Robert Mach, Kyle C. Wilcox, Sjoerd Finnema, Magnus Schou, Andrea Varrone*.

Corresponding authors:

*Dinahlee Saturnino Guarino, e-mail: dinahlee.saturninoguarino@pennmedicine.upenn.edu

*Andrea Varrone, e-mail: andrea.varrone@ki.se

General:

Chemicals and solvents were obtained from commercial sources and were used without further purification. KAC-50.1 was prepared by the previously reported method.[1] NMR spectra were recorded on Bruker 400 or 500 MHz AVANCE III system using standard Bruker pulse sequences. Experiments were run in CD_3OD, $CDCl_3$ or D_6-DMSO at 25 °C. ^{1}H NMR chemical shifts are referenced relative to the residual solvent peak at 3.33, 7.26, or 2.50 ppm, respectively, and ^{13}C NMR chemical shifts are referenced to 77.0 ppm for $CDCl_3$ or 39.5 for D_6-DMSO. Flash column chromatography was carried out using prepacked silica gel columns supplied by Biotage and using a Biotage automated flash system with UV detection. Reactions with tritium gas were performed on a RC Tritec tritium manifold. Analytical HPLC was carried out using an agilent 1100 series and LCMS analysis was carried out using a agilent 1200 series with a Waters 4.6 × 100 mm Xselect CSH C18 3.5 µ, 5 to 95% MeCN/water 0.2% formic acid pH 3 for 1.85 min then isocratic elution for 0.1 min with mass detection using a QDA mass detector. The molar activities of the products were determined by LC/MS using Isopat2 to deconvolute the MS signals.[2] Liquid scintillation counting was performed with a Beckman LS 6500 scintillation counter.

1-phenyl-4-vinyl-1H-1,2,3-triazole

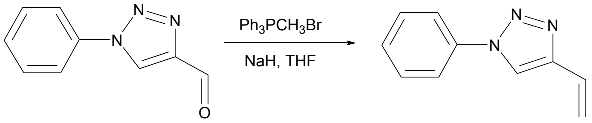

The procedure of Boechat was followed for this reaction.[3] A slurry of NaH (0.680 g, 17.0 mmol) in THF (100 mL) under N_2 was stirred as methyltriphenylphosphonium bromide (12.41 g, 34.74 mmol) was added portionwise. After 2 hours, 1-phenyl-1H-1,2,3-triazole-4-carbaldehyde (1.28 g, 7.39 mmol) was added, and the mixture was stirred for another 2 hours. The reaction mixture was added to water (230 mL) and the resulting suspension was extracted with EtOAc (2 x 200

mL). The organics were combined and dried over a phase separator cartridge and then concentrated to dryness. The residue was purified by flash chromatography (5% to 100% of ethyl acetate in heptane). Product containing fractions were combined and concentrated to afford 1-phenyl-4-vinyl-1H-1,2,3-triazole as a white solid (0.850 g, 67%).

LCMS (M+1): 172.1.

^{1}H NMR (500 MHz, $CDCl_3$) δ 7.94 (s, 1H), 7.75 (m, 2H), 7.54 (m, 2H), 7.45 (m, 1H), 6.80 (dd, *J* = 17.8, 11.2 Hz, 1H), 6.02 (dd, *J* = 17.7, 1.3 Hz, 1H), 5.43 (dd, *J* = 11.2, 1.2 Hz, 1H).

(E)-4-(2-(1-phenyl-1H-1,2,3-triazol-4-yl)vinyl)phenol (AZ14226351)

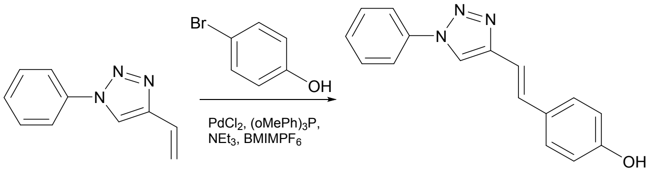

A modification of the procedure of Diaz-Ortiz was used.[1] A solution of palladium(II) chloride (17 mg, 0.09 mmol), tri-o-tolylphosphane (57 mg, 0.19 mmol) and 1-butyl-3-methyl-1H-imidazol-3-ium hexafluorophosphate(V) (770 µl, 3.7 mmol) were heated at 80 °C for 5 min in an oil bath after which 1-phenyl-4-vinyl-1H-1,2,3-triazole (400 mg, 2.34 mmol), triethylamine (490 µl, 3.50 mmol) and 4-bromophenol (404 mg, 2.34 mmol) were added at 80 °C. The vial was purged with N2 and was then sealed and heated at 150 °C for 24 min in a Biotage microwave reactor. The dark mixture was partitioned between water and ethyl acetate, and the aqueous layer was extracted twice (2 x 50 mL EtOAc). The combined organic phases were dried using a phase separator cartridge, and the solvent was removed. The black residue was purified by flash chromatography (70% to 100% of EtOAc in N-heptane) amd the product containing fractions were combined and concentrated to afford a yellow solid (280 mg, 80% pure by UV). The sample was further purified by preparative HPLC (10-80% MeCN in H_2O with 0.1% TFA), and the product containing fractions were combined. The combined column fractions were concentrated to approx half volume at reduced pressure and then lyophilized to afford (E)-4-(2-(1-phenyl-1H-1,2,3-triazol-4-yl)vinyl)phenol (0.140 g, 22.76 %) as a white solid. NMR still shows small amount of ethyl acetate present which was used to transfer the material after-freeze drying.

LCMS (M+1): 264.2
^{1}H NMR (500 MHz, DMSO) δ 9.63 (s, 1H), 8.91 (s, 1H), 7.91 (m, 2H), 7.61(m, 2H), 7.50 (m, 1H), 7.43(m, 2H), 7.28 (d, *J* = 16.5 Hz, 1H), 7.02 (d, *J* = 16.5 Hz, 1H), 6.80(m, 2H).
^{13}C NMR (126 MHz, DMSO) δ 158.1, 147.2, 137.1, 131.0, 130.4, 129.2, 128.4, 128.0, 120.4, 119.7, 116.1, 114.0.

diethyl (3-iodo-4-methoxybenzyl)phosphonate

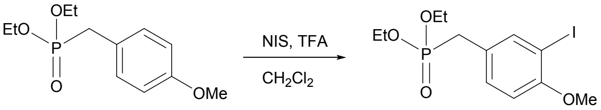

A slurry of diethyl (4-methoxybenzyl)phosphonate (520 mg, 2.01 mmol) and 1-iodopyrrolidine-2,5-dione (601 mg, 2.67 mmol) in CH_2Cl_2 (5 mL) and TFA (1 mL) was degassed with N_2 for 3 min and was then capped and stirred for 2h. The slurry was concentrated to dryness to give a yellow solid which was purified by silica gel chromatagraphy (10 to 100% Heptane-EtOAc). The product containing fractions were combined to give 566 mg of a yellow oil.[4]

LCMS (M+1): 385.1.

(E)-4-(3-iodo-4-methoxystyryl)-1-phenyl-1H-1,2,3-triazole

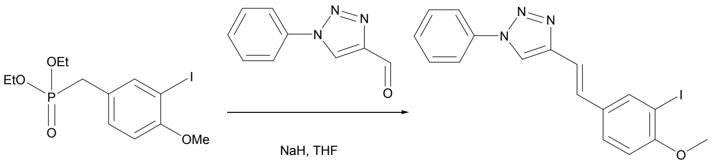

A solution of diethyl (3-iodo-4-methoxybenzyl)phosphonate (280 mg, 0.73 mmol) and 1-phenyl-1H-1,2,3-triazole-4-carbaldehyde (150 mg, 0.87 mmol) in THF (5 mL) at 0 °C was stirred as NaH (40 mg, 1.00 mmol) was added. The reaction was stirred 30 min under a N_2 stream while warming to rt. Then the vial was capped and stirred over the weekend. A second batch of 40 mg of NaH was added and the solution was heated at 50 C for 3 h. Partitioned the reaction mixture between, 50 mL of water and 50 mL of EtOAc. The laters were separated and the organic phase extracted with 50 mL of sat aq. NaHCO3, 50 mL of 2.5 M NaOH, 25 mL water, and 25 mL of sat aq NaCl. The organic solution was dried over $MgSO_4$,and the organic layer was filtered and concentrated to afford 130 mg of the crude reactions mixture. The crude mixture was purified by prepartive HPLC (Waters XBridge Prep C18 5µ OBD 19x150mm, 25-85% MeCN in aq. TFA 0,1% over 20 min. at a flow rate of 20mL/min) to give 70 mg of a pale yellow solid.

LCMS (M+1): 404.1. (approx 4% Z isomer is present)

^{1}H NMR (400 MHz, CDCl3) δ 7.97 (d, 2H), 7.71 – 7.79 (m, 2H), 7.5 – 7.59 (m, 2H), 7.45 (tt, 2H), 7.30 (d, 1H), 7.00 (d, 1H), 6.82 (d, 1H), 3.90 (s, 3H).
^{13}C NMR (100 mHz): 158.0, 146.7, 137.3, 137.0, 131.5,129.8, 129.3, 238.8, 128.1, 120.4, 118.1, 115.3, 110.9 86.4, 56.5.

(E)-4-(3-[^{3}H]-4-methoxystyryl)-1-phenyl-1H-1,2,3-triazole ([^{3}H]KAC-50.1)

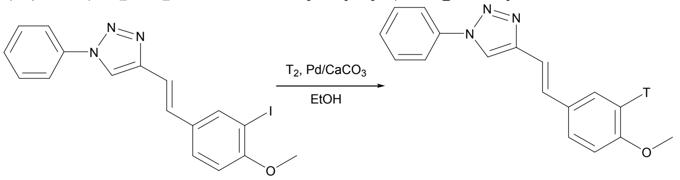

(E)-4-(3-iodo-4-methoxystyryl)-1-phenyl-1H-1,2,3-triazole (1.6 mg, 3.97 µmol), Pd on $CaCO_3$ (2.4 mg, 0.90 µmol) and ethanol (absolute, 99.5%) (0.6 mL) were mixed in the reaction flask, the mixture was de-gassed 3x via freeze-thaw methodology. Tritium (0.903 mg, 0.15 mmol, 160 GBq) was added, the mixture was stirred for 2.5h. Unreacted tritium gas (89 GBq) was recovered, and the volatiles were removed by a stream of N_2. MeOH was added twice and removed under an N_2 stream. The residue was dissolved in 1 mL of MeOH and was filtered, and

the eluant was concentrated to dryness. The residue was dissolved in 15 mL of EtOH to give 405 MBq of crude product which was purified by of the reverse phase chromatography (Waters XBridge Prep C18 5µ OBD 19x150 mm, 20-85% MeCN in aq. TFA 0.1% over 20 min. at a flow rate of 20 mL/min. Product containing fractions were combined and concentrated to dryness and the residue was taken up in 2 mL of EtOH to give 134 MBq.

LC-Rad: 99.2% (Waters Xbridge C18 3.5 µm, 4.6 x100 mm, 5% for 3 min then 5 to 95% over 25 min and 5 min at 95% of MeCN-0.1 M Formic acid in water (pH 3).
LCMS (M+1): 278 (15.9%), 279 (3.6%), 280 (100%), 281 (19.1%), 282 (2.4%).
Molar Activity: 910 kBq/nmol.
^{1}H NMR (CD_3OD, 500 MHz): 8.61 (s, 1H), 7.88 (m, 2H), 7.60 (dd, 2H), 7.50 (m, 3H), 7.36 (d, 1H), 7.05 (d, 1H), 6.94 (d, 1.3 H).
^{3}H NMR: (CD_3OD, 550 MHz): 6.94 (s)

HPLC and Radio-HPLC plots of [^{11}C]KAC-50.1

a)

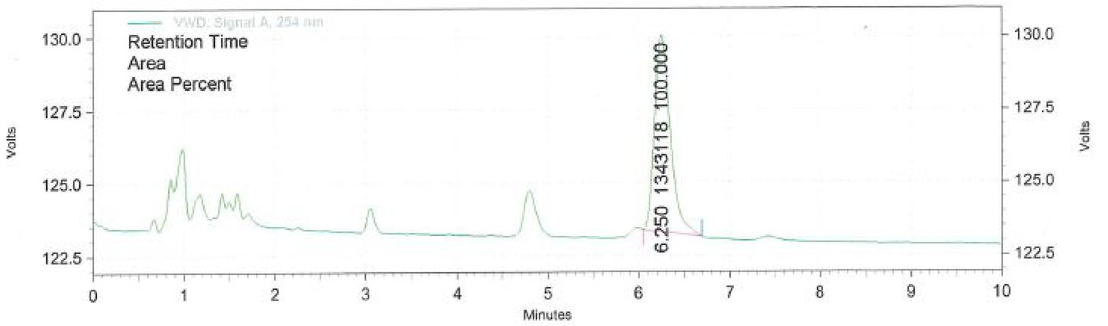

b)

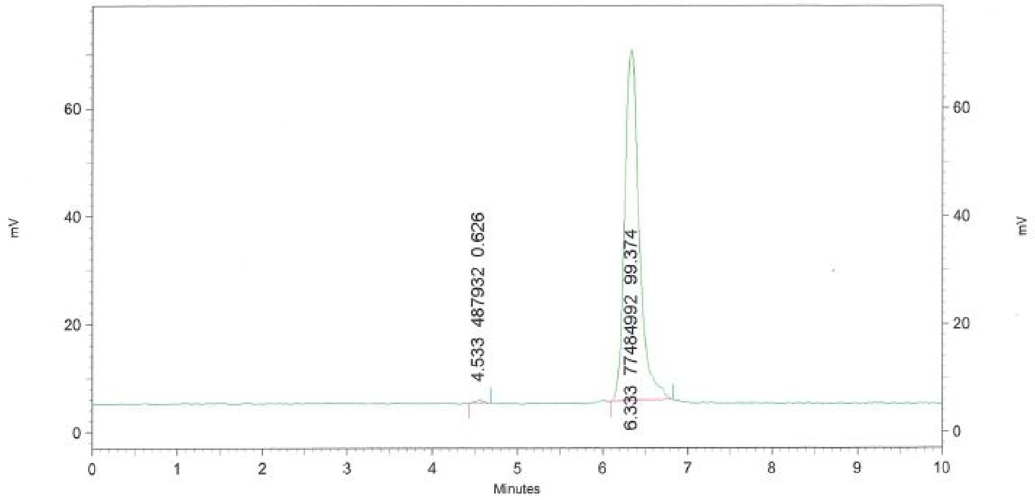

c)

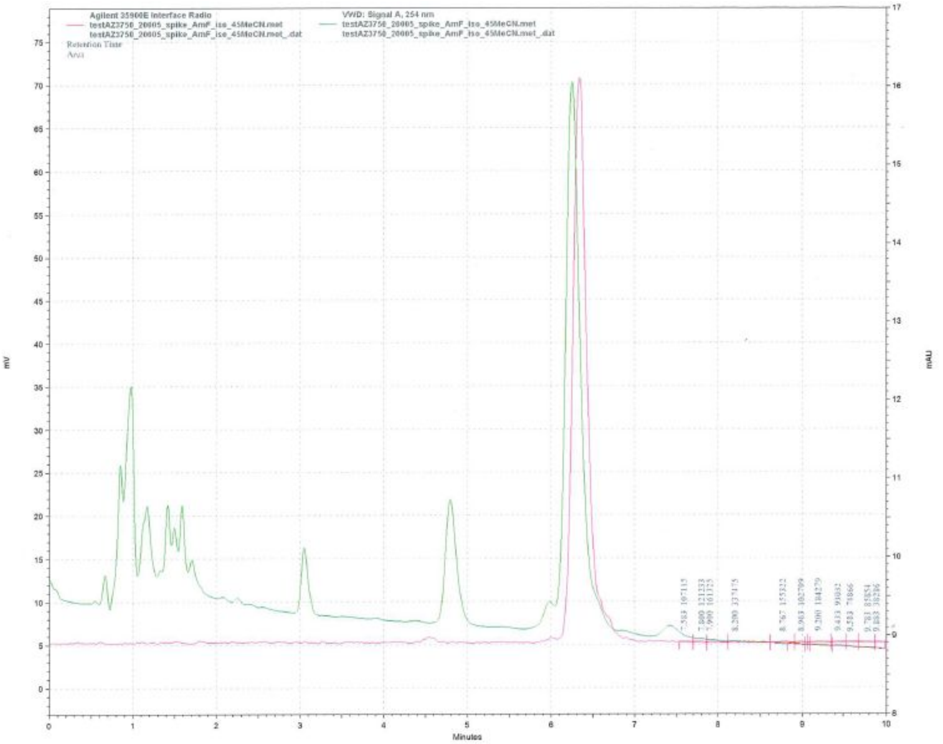

Figure S1. Analytical HPLC chromatograms of [^{11}C]AZ14203750 coinjected with reference AZ14203750. XBridge C18 column (130Å, 5 µm, 4.6 mm X 150 mm), λ = 254 nm, MeCN/AmF (aq. 0.1M) 50:50, 2.0 mL/min. a) UV-trace. b) Radioactivity trace. c) Superimposition of UV (green) and radioactivity (red) trace.

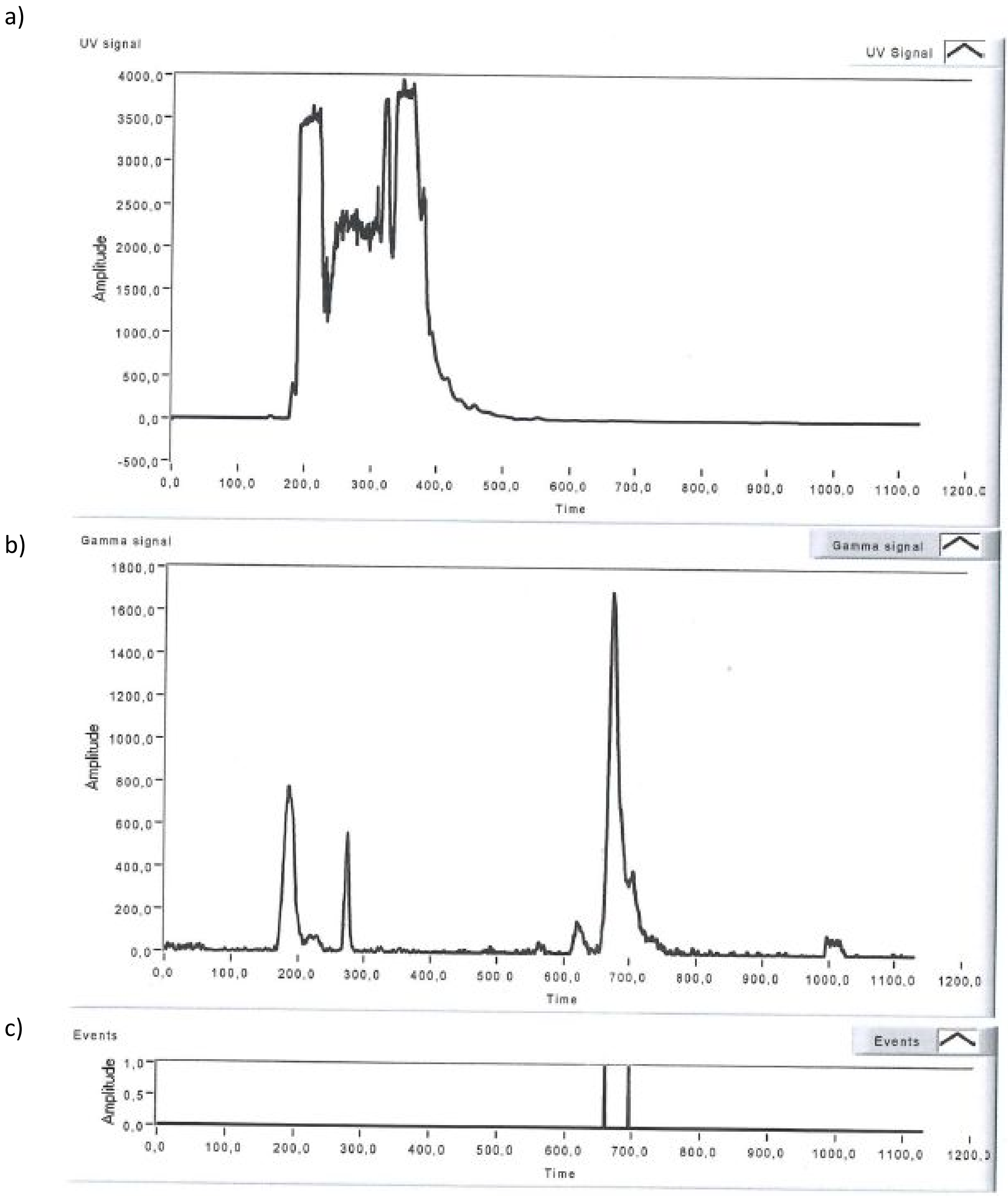

Figure S2. Semipreparative HPLC chromatogram of [^{11}C]AZ14203750. XBridge C18 semipreparative column (130Å, 5 µm, 10 mm X 250 mm), λ = 254 nm, MeCN/AmF (aq. 0.1M) 54:46, 6.0 mL/min. a) UV-trace. b) Radioactivity trace. c) Collected fraction.

High throughput screening using in *vitro* binding assays with α-syn fibrils and maxi TMA autoradiography

Compounds structurally related to Tg-1-90B from internal library were selected and screened in vitro using competition binding with [^{3}H]Tg-1-90B in the presence of α-synuclein fibrils (Fig. S3).

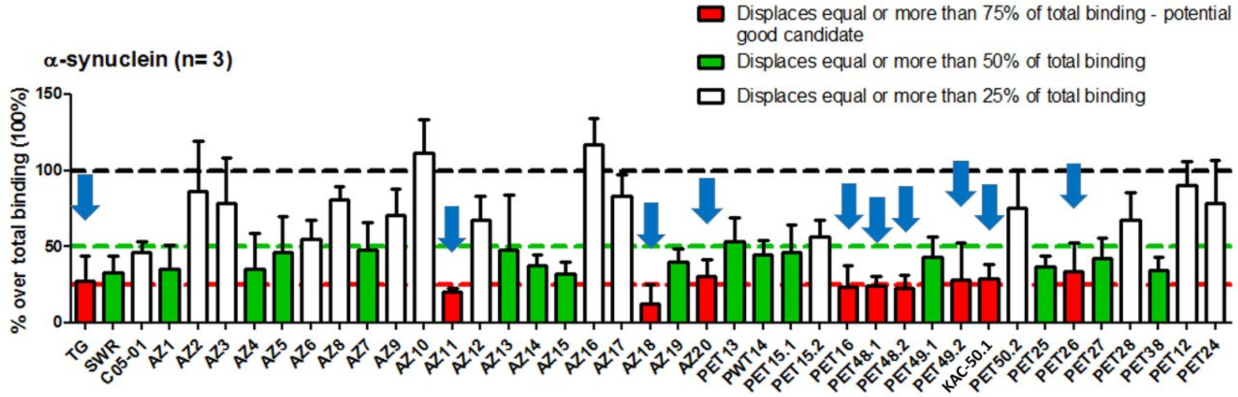

Figure S3. Binding screening using [^{3}H]TG-1-90B and α-synuclein fibrils. SWR corresponds to BF-2846. In red the compounds displaying ≥75% of total binding.

In a separate set of experiments the selected compounds (red columns) were screened on TMAs from MSA, AD and CAA cases in competition with [^{3}H]Tg-1-90B (Fig S4 and 5). In MSA cases PET compounds 50.1, 48.1 and 16 displaced at the same extent as Tg-1-90B, suggesting similar affinity (Fig. S4). In CA and AD cases PET compounds 50.1, 48.1 and 16 in correspondence of Aβ deposits displaced less than Tg-1-90B, suggesting a higher selectivity over Aβ compared to Tg-1-90B (Fig. S4).

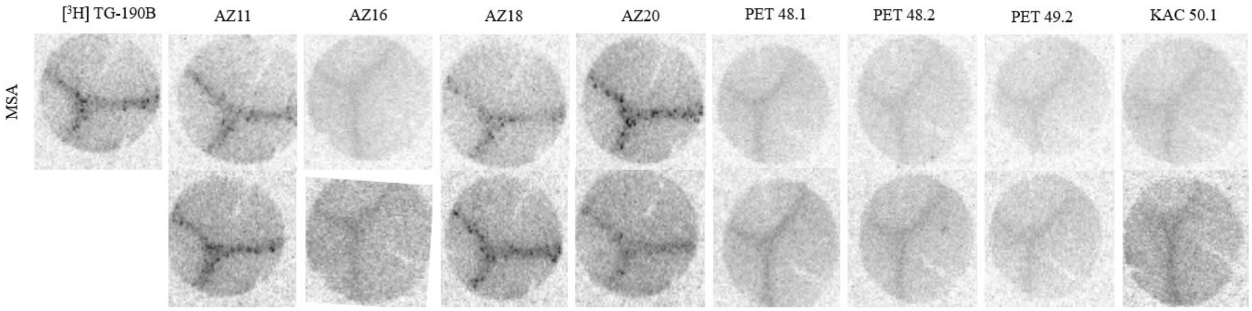

Figure S4. ARG based inhibition assay using compounds AZ11, AZ16, AZ18, AZ20, PET48.1, PET 48.2, PET 49.2 and KAC 50.1 in two concentrations (100 nM first row and 50 nM second row) as competitors of [^{3}H]TG-1-90B in TMAs sections of an MSA case.

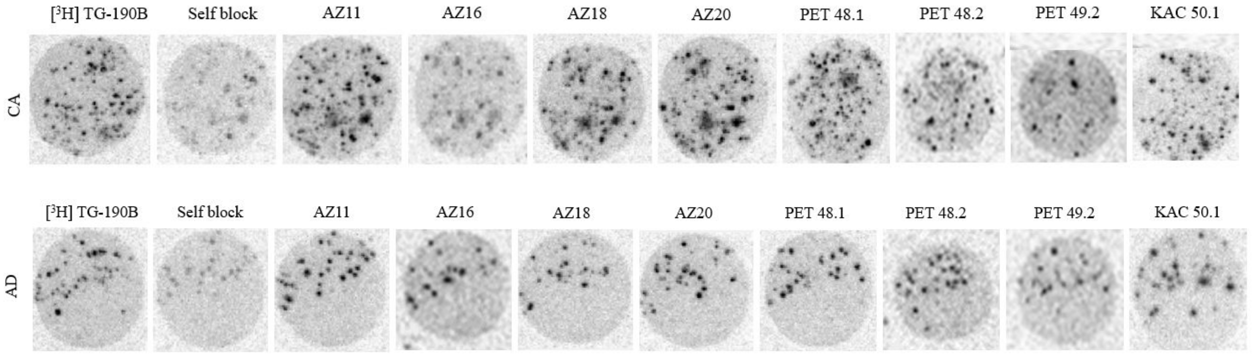

Figure S5. ARG based inhibition assay in Cerebral amyloid angiopathy (CA) and Alzheimer's disease (AD) pathologies using compounds AZ11, AZ16, AZ18, AZ20, PET48.1, PET 48.2,PET 49.2 and KAC 50.1 as competitors at 50nM concentration.

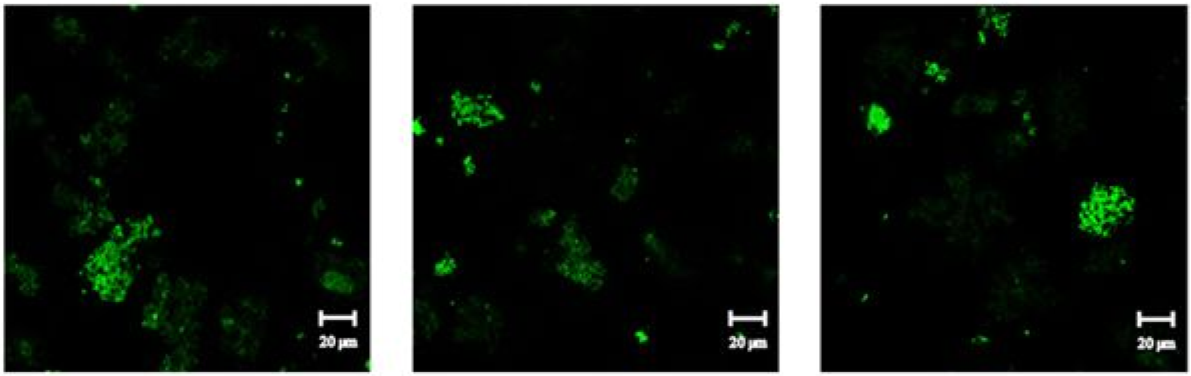

Figure S6. Fluorescence images of α-synuclein fibrils by using 10 μM of ThT dye.

[^{3}H]PiB counterscreening in AD tissue homogenates

Fresh frozen samples of human frontal cortex were obtained from the Brain and Body Donation Program of the Banner Sun Health Research Institute and were validated by the vendor by immunohistochemistry to be free of tau, amyloid, and alpha synuclein (control donor) or free of tau and alpha synuclein (Braak IV donor). Samples were homogenized in PBS at a concentration of 10 mg wet weight per mL buffer and stored at -80 °C. Saturation assays were conducted in 96 well plates containing 0.4 mg membrane per well and tritiated ligands across a range of half-log dilutions from 100 nM (PiB) or 126 nM (PET 50.1). Nonspecific binding was determined by the addition of 10 µM (PiB) or 4 µM (PET 50.1) cold compounds. Reaction plates were incubated at room temperature for 90 minutes and filtered onto PEI-coated GF/C filter plates using a Perkin-Elmer cell harvester. Filters were washed using 400 mL ice-cold PBS and allowed to dry before adding 40 µL Microscint-20 per well for counting on a Perkin-Elmer MicroBeta-2 plate reader. A standard curve was prepared using each tritiated tool compound by adding activity directly to a cell harvester filter plate, allowing the samples to dry, and adding scintillation fluid as described above. Data was imported into Microsoft Excel and CPM values were converted to fmol/mg membrane (wet weight) using the standard curves. Standardized values were plotted in GraphPad Prism and fitted using a 1-site Total and Nonspecific Binding model.

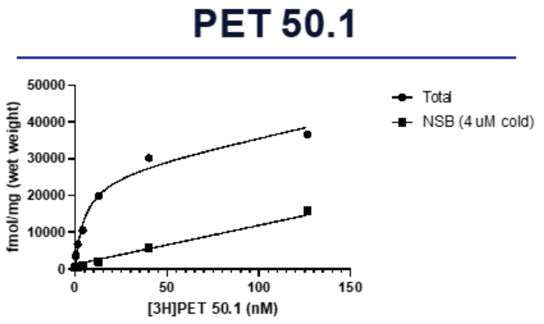

Best-fit values	
Bmax	24892
Kd	5.503
NS	106.2
Background	1250
Std. Error	
Bmax	960.5
Kd	0.8286
NS	6.463
Background	315.8

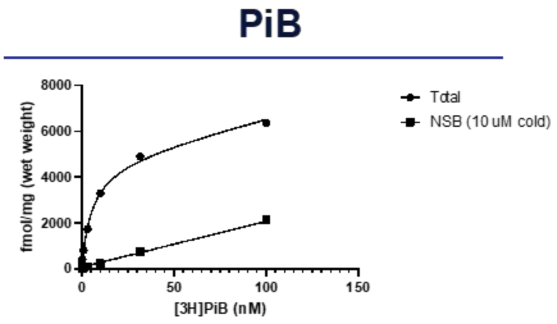

Best-fit values	
Bmax	4675
Kd	5.561
NS	19.87
Background	82.18
Std. Error	
Bmax	91.25
Kd	0.3910
NS	0.7018
Background	23.96

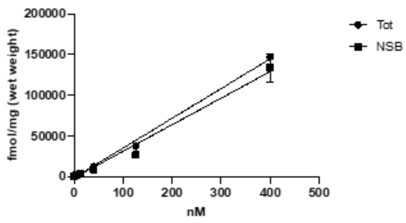

Best-fit values	
Bmax	3.398e+016
Kd	8.719e+014
NS	326.0
Background	-1314
Std. Error	
Bmax	2.755e+016
Kd	5.016e+014
NS	14.38
Background	1351

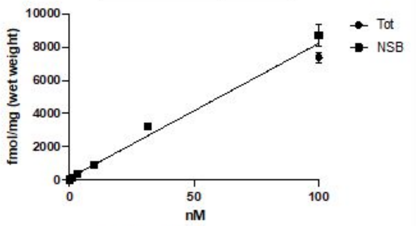

Best-fit values	
Bmax	~ 229.3
Kd	~ 1.594e+015
NS	81.00
Background	124.8
Std. Error	
Bmax	~
Kd	~
NS	6.442
Background	164.1

Figure S7. Saturation binding experiments with [^{3}H]KAC 50.1 and [^{3}H]PiB in brain homogenates enriched with Aβ (no tau) from Braak IV (top row) and amyloid-free control donor (bottom row).

In vitro Autoradiography in TMAs

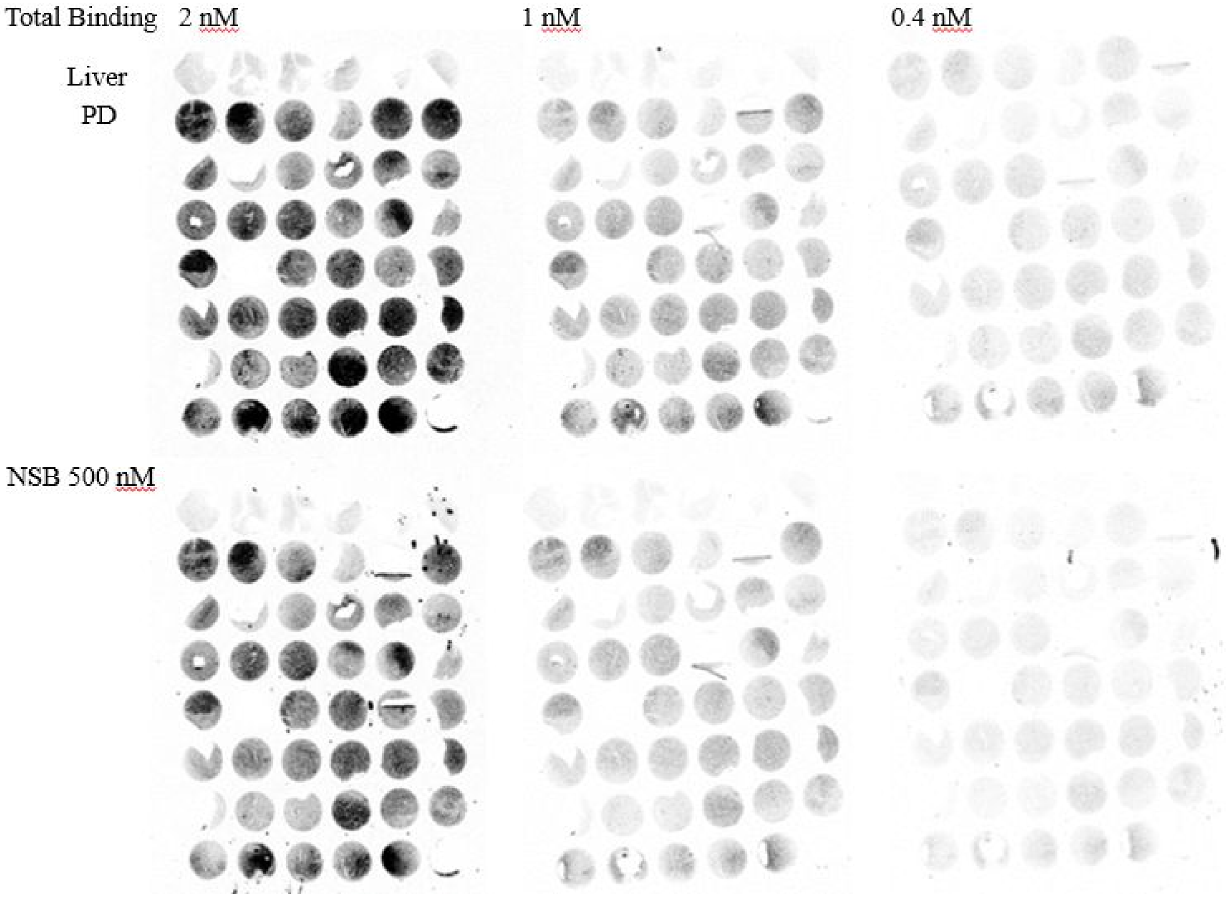

Figure S8. In vitro autoradiography of [^{3}H]KAC-50.1 in TMA 3 in the presence of 2 nM, 1 nM and 0.4nM of radiolabeled compound. Non-specific binding was determined in the presence of 500 nM of KAC-50.1.

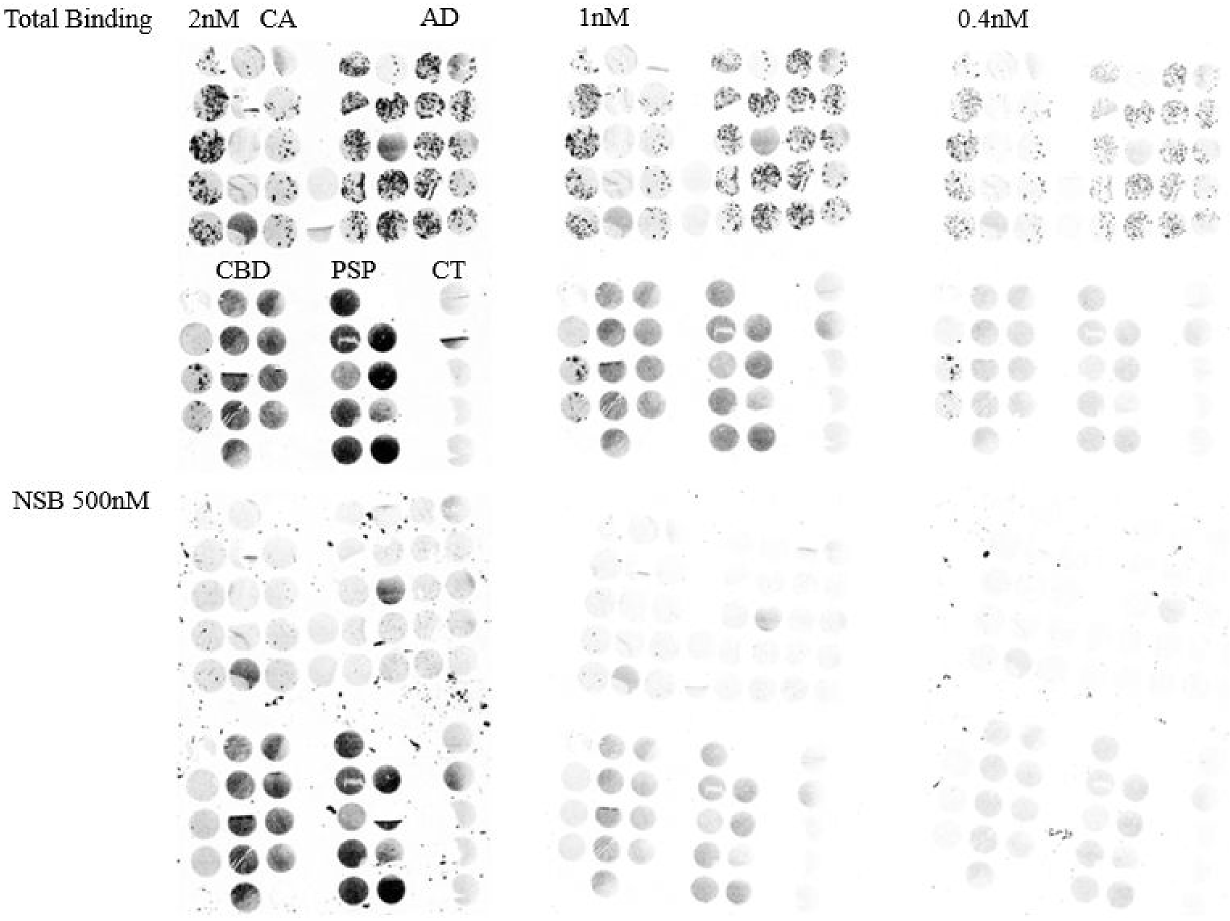

Figure S9. In vitro autoradiography of [^{3}H] KAC-50.1 in TMA 2 in the presence of 2 nM, 1 nM and 0.4 nM of radiolabeled compound. Non-specific binding was determined in the presence of 500 nM of KAC-50.1.

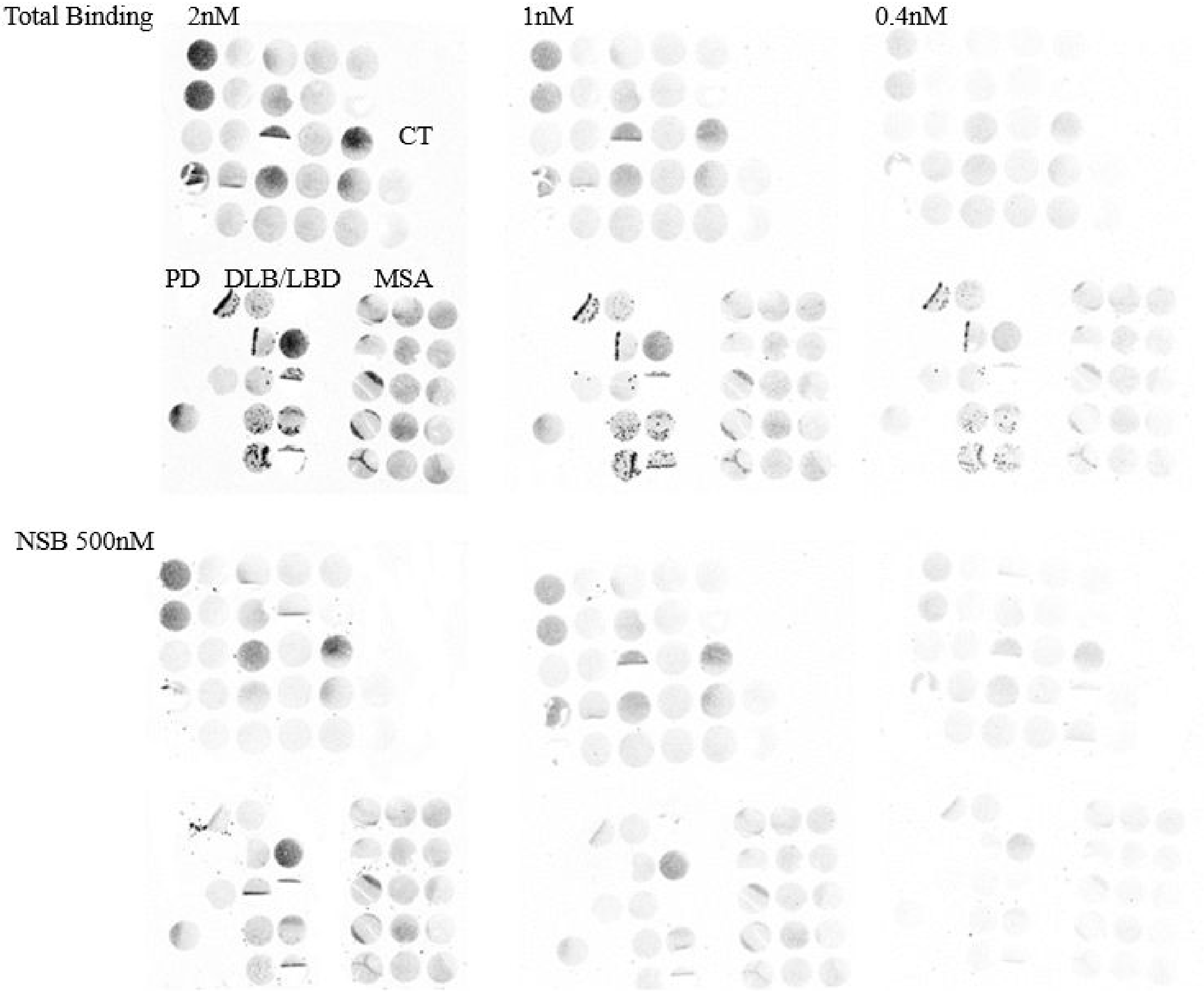

Figure S10. In vitro autoradiography of [^{3}H]KAC-50.1 in TMA 1 in the presence of 2 nM, 1 nM and 0.4 nM of radiolabeled compound. Non-specific binding was determined in the presence of 500 nM of KAC-50.1.

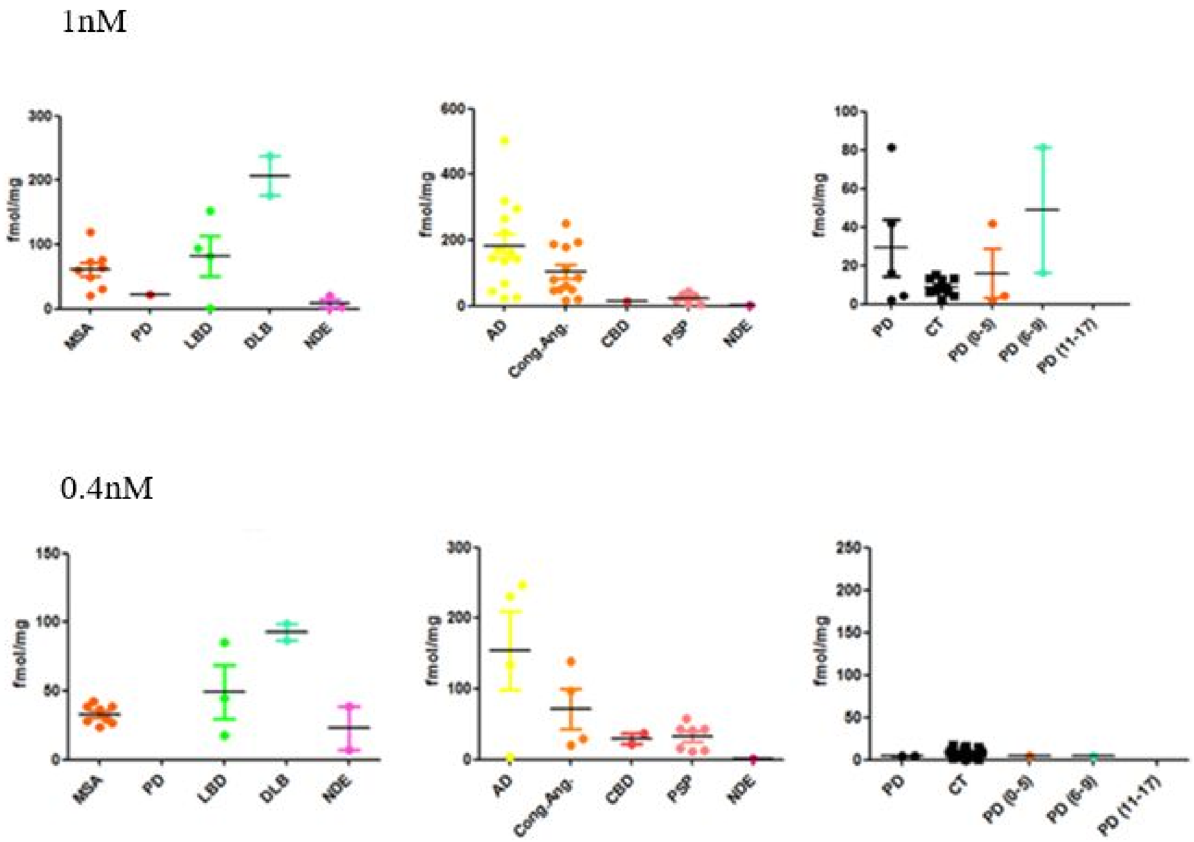

Figure S11. Assessment of specific binding of [^{3}H]KAC-50.1 to α-syn, A-β and tau aggregates in human tissue microarrays sections using 1 nM and 0.4 nM of radiolabeled compound.

Non-human primate PET imaging with [^{11}C]KAC-50.1

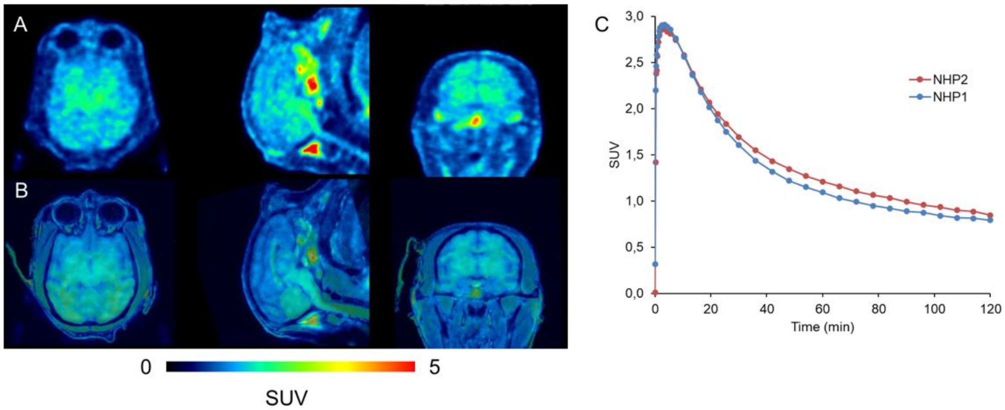

Figure S12. A) [^{11}C]KAC-50.1 mean image from 5 to 123 min in NHP2 and B) overlaid on individual MRI. C) Whole-brain time-activity curves of [^{11}C]KAC-50.1 in both NHPs.

1. Díaz-Ortiz, Á.; Prieto, P.; de Cózar, A.; Cebrián, C.; Moreno, A.; de la Hoz, A., Microwave-Controlled Preparation of Alkenyl-(1H)-1,2,4-triazoles: First Heck Reaction on a (1H)-1,2,4-Triazole Moiety. *Australian Journal of Chemistry* **2009,** *62* (12), 1600-1606.
2. Gruber, C. C.; Oberdorfer, G.; Voss, C. V.; Kremsner, J. M.; Kappe, C. O.; Kroutil, W., An Algorithm for the Deconvolution of Mass Spectrosopic Patterns in Isotope Labeling Studies. Evaluation for the Hydrogen-Deuterium Exchange Reaction in Ketones. *J Org Chem* **2007,** *72*, 5778-5783.
3. Boechat, N.; Ferreira, V. F.; Ferreira, S. B.; Ferreira, M. d. L. G.; da Silva, F. d. C.; Bastos, M. M.; Costa, M. d. S.; Lourenço, M. C. S.; Pinto, A. C.; Krettli, A. U.; Aguiar, A. C.; Teixeira, B. M.; da Silva, N. V.; Martins, P. R. C.; Bezerra, F. A. F. M.; Camilo, A. L. S.; da Silva, G. P.; Costa, C. C. P., Novel 1,2,3-Triazole Derivatives for Use against Mycobacterium tuberculosis H37Rv (ATCC 27294) Strain. *Journal of Medicinal Chemistry* **2011,** *54* (17), 5988-5999.
4. Hart, D. J.; Leroy, V.; Merriman, G. H.; Young, D. G. J., C-Aryl glycosides: electrophile-initiated cyclizations of 6-aryl-5-hexen-2-ols. *The Journal of Organic Chemistry* **1992,** *57* (21), 5670-5680.
